# Galectin-9 and IL-21 Mediate Cross-regulation between Th17 and Treg Cells during Acute Hepatitis C

**DOI:** 10.1371/journal.ppat.1003422

**Published:** 2013-06-20

**Authors:** Hassen Kared, Thomas Fabre, Nathalie Bédard, Julie Bruneau, Naglaa H. Shoukry

**Affiliations:** 1 Centre de Recherche du Centre Hospitalier de l'Université de Montréal (CRCHUM), Hôpital St-Luc, Montréal, Québec, Canada; 2 Département de microbiologie et immunologie, Université de Montréal, Montréal, Québec, Canada; 3 Departement de médecine familiale, Université de Montréal, Montréal, Québec, Canada; 4 Département de médecine, Faculté de médecine, Université de Montréal, Montréal, Québec, Canada; University of Washington, United States of America

## Abstract

Loss of CD4 T cell help correlates with virus persistence during acute hepatitis C virus (HCV) infection, but the underlying mechanism(s) remain unknown. We developed a combined proliferation/intracellular cytokine staining assay to monitor expansion of HCV-specific CD4 T cells and helper cytokines expression patterns during acute infections with different outcomes. We demonstrate that acute resolving HCV is characterized by strong Th1/Th17 responses with specific expansion of IL-21-producing CD4 T cells and increased IL-21 levels in plasma. In contrast, viral persistence was associated with lower frequencies of IL-21-producing CD4 T cells, reduced proliferation and increased expression of the inhibitory receptors T cell immunoglobulin and mucin-domain-containing-molecule-3 (Tim-3), programmed death 1 (PD-1) and cytotoxic T-lymphocyte antigen 4 (CTLA-4) on HCV-specific CD8 T cells. Progression to persistent infection was accompanied by increased plasma levels of the Tim-3 ligand Galectin-9 (Gal-9) and expansion of Gal-9 expressing regulatory T cells (Tregs). *In vitro* supplementation of Tim-3^high^ HCV-specific CD8 T cells with IL-21 enhanced their proliferation and prevented Gal-9 induced apoptosis. siRNA-mediated knockdown of Gal-9 in Treg cells rescued IL-21 production by HCV-specific CD4 T cells. We propose that failure of CD4 T cell help during acute HCV is partially due to an imbalance between Th17 and Treg cells whereby exhaustion of both CD4 and CD8 T cells through the Tim-3/Gal-9 pathway may be limited by IL-21 producing Th17 cells or enhanced by Gal-9 producing Tregs.

## Author Summary

In this study, we investigated the mechanisms underlying failure of the CD4 helper T cell response during acute hepatitis C infection. We demonstrate that this failure is primarily due to loss of IL-21-producing CD4 T cells in individuals who progress towards chronic infection. This is accompanied by exhaustion of virus-specific cytotoxic CD8 T cells through upregulation of the exhaustion markers Tim-3, PD-1 and CTLA-4, higher plasma levels of the Tim-3 ligand Galectin-9 (Gal-9) and increased frequency of Gal-9 producing regulatory T cells (Tregs). *In vitro* supplementation with IL-21 rescued HCV-specific CD8 T cells from Gal-9 induced apoptosis. Blocking Gal-9 expression in Tregs restored IL-21 production by virus-specific CD4 helper T cells. Altogether, our results suggest that failure of CD4 T cell help during acute HCV may be partially meditated by an imbalance between IL-21-producing CD4 T cells and Treg cells whereby exhaustion of both CD4 and CD8 T cells through the Tim-3/Gal-9 pathway is counteracted by IL-21.

## Introduction

The outcome of acute hepatitis C virus (HCV) infection towards spontaneous resolution or persistent viremia is dictated by the magnitude, breadth and quality of the virus-specific CD4 and CD8 T cell responses [1], [2]. The essential role of CD4 helper T cells in mediating spontaneous viral clearance was demonstrated by several observations. First, the loss of CD4 helper T cell proliferative responses during acute HCV was associated with viral recurrence and the development of chronic infection [3], [4]. Second, broad HCV-specific CD4 T cell responses are induced early in most acutely infected individuals but they undergo progressive loss of IL-2 production and diminished proliferation as infections progress towards viral persistence [5]–[8]. Third, CD4 T cell depletion in the chimpanzee model of HCV infection led to persistent low level viremia, the loss of CD8 function and the development of escape mutations in targeted CD8 cytotoxic T lymphocyte (CTL) epitopes [9]. These observations strongly suggest that CD4 helper T cells are critical in sustaining the functions of HCV-specific CD8 T cells. However, the underlying helper signals and the mechanisms of CD4 T cell failure remain elusive.

T cell exhaustion has been proposed as a mechanism underlying the dysfunction of HCV-specific CD4 and CD8 T cells during acute infection. The over-expression of inhibitory receptors like T cell immunoglobulin and mucin-domain-containing-molecule-3 (Tim-3), programmed death 1 (PD-1) and cytotoxic T-lymphocyte antigen 4 (CTLA-4) and 2B4 was observed on HCV-specific CD8 T cells in the blood and liver of individuals developing chronic HCV infection (reviewed in [10]). Blockade of these inhibitory pathways restored proliferation and cytokine production by HCV-specific CTLs [10]. The differential level of expression of these inhibitory receptors on virus-specific T cells and their respective ligands in certain tissues may contribute to various levels of exhaustion. For example, higher levels of exhaustion and apoptosis are observed in the liver where greater levels of the PD-1 ligand-1 (PDL-1) and the Tim-3 ligand Galectin-9 (Gal-9) are expressed [11]–[16]. Using MHC class II tetramers, Raziorrouh et al. have observed the increased expression of PD-1 and CTLA-4 on virus-specific CD4 T cells from patients with chronic HCV infection [17]. Blocking the PD-1 pathway restored the proliferation of HCV-specific CD4 helper T cells and the production of the Th1 cytokines interferon-gamma (IFN-γ) and tumor necrosis factor alpha (TNF-α) [17]. Whether this exhausted phenotype affects the production of other helper cytokines and mediators of CD4 T cell help was not investigated.

Other possible mechanisms of T cell failure include inhibition of proliferation by Tregs or imbalance between the different CD4 helper T cell subsets (e.g. Th1, Th2, Th17) during the progression of HCV infection. Increased Treg frequencies were observed in chronic HCV infection [18], [19]. Tregs can have a direct inhibitory effect on virus-specific CD4 and CD8 T cells through production of the immuno-modulatory cytokines IL-10 and transforming growth factor beta (TGF-β) [17] or through expression of Gal-9 as observed during human immunodeficiency virus (HIV) infection [20]. Another subset that has become increasingly important is Th17 cells that produce a variety of cytokines including IL-17A, IL-21 and IL-22. IL-17A-producing CD4 and CD8 T cells are highly enriched in the liver [21]–[23] and HCV-specific Th17 cells were detected in the peripheral blood during chronic HCV infection [24]. In addition, a temporal association was observed between increased virus-specific IL-17 responses and spontaneous recovery from recurrent hepatitis C in a liver transplant recipient [25]. Th17 cells were also associated with the control of several bacterial and viral infections [26] and are a potential source of IL-21 that has been recently identified as a major helper cytokine during chronic viral infection. Studies in the mouse model of lymphocytic choriomeningitis virus (LCMV) have demonstrated that IL-21 is required to sustain proliferation and effector functions of virus-specific CD8 T cells [27]–[29]. Similarly, data from HIV-infected individuals have demonstrated that IL-21 is associated with viral control and slower disease progression [30]–[32]. However, the role of Th17 cells and that of IL-21 as a helper cytokine during acute HCV infection were not studied.

In this study, we performed a longitudinal analysis of CD4 helper T cell function and interaction between CD4 and CD8 T cells during acute HCV infection in individuals with spontaneous resolution *vs* persistent viremia. We specifically focused on IL-21 producing Th17 cells and the means by which Tregs control helper activity of CD4 T cells. We demonstrate a delicate balance between IL-21-producing Th17 cells and Gal-9 producing Tregs. This balance may favor either exhaustion or survival of HCV-specific CD4 and CD8 T cells during acute HCV infection.

## Results

### Preferential expansion of virus-specific IL-21-producing Th17 cells during acute resolving HCV

The first goal of this study was to longitudinally examine and compare the magnitude and functional profile of HCV-specific CD4 T cells during acute HCV infections with different outcomes. However, the very low frequency of virus-specific CD4 T cells and the limited availability of HCV MHC class II tetramers prevented us from performing direct *ex vivo* analysis of these cells. Thus, we developed an assay combining carboxyfluorescein succinimidyl ester (CFSE) proliferation and intracellular cytokine staining (ICS) to determine the cytokine profile of HCV-specific CD4 T cells (Supplementary Figure S1A). This is a qualitative rather than a quantitative assay that provides a general overview of the quality and function of HCV-specific CD4 T cells during different stages of infection. Peripheral blood mononuclear cells (PBMCs) were depleted of CD25^+^ cells before stimulation to limit inhibition of antigen-specific proliferation by Tregs and facilitate detection of HCV-specific T cells activated during the *in vitro* stimulation. Treg-depleted-PBMCs from HCV patients were labelled with CFSE and stimulated with HCV recombinant proteins in a standard 6 day CFSE proliferation assay as described in Materials and Methods and Supplementary Figure S1A. Virus-specific CD4 T cells were identified by the dilution of CFSE staining coupled with the up-regulation of CD25, used as an early activation marker. FACS plots from a representative proliferation assay are presented in Supplementary Figure S1B. At the end of the proliferation on day 6, cells were stimulated with phorbol 12-myristate 13- acetate (PMA) and ionomycin in a standard ICS assay to reveal their cytokine profile by gating on proliferating (CFSE^low^) cells. Due to the fact that proliferation and PMA/ionomycin induce down-modulation of CD4, HCV-specific CD4 T cells were defined by gating on CD3^+^CD8^neg^ T cells and examining the cytokines produced by CFSE^low^ cells. We monitored the production of the Th1 cytokine: IFN-γ and TNF-α as well as the Th17 cytokines IL-17A and IL-21. Representative FACS analysis plots for the ICS assay are presented in Supplementary Figure S2.

**Figure 1:**
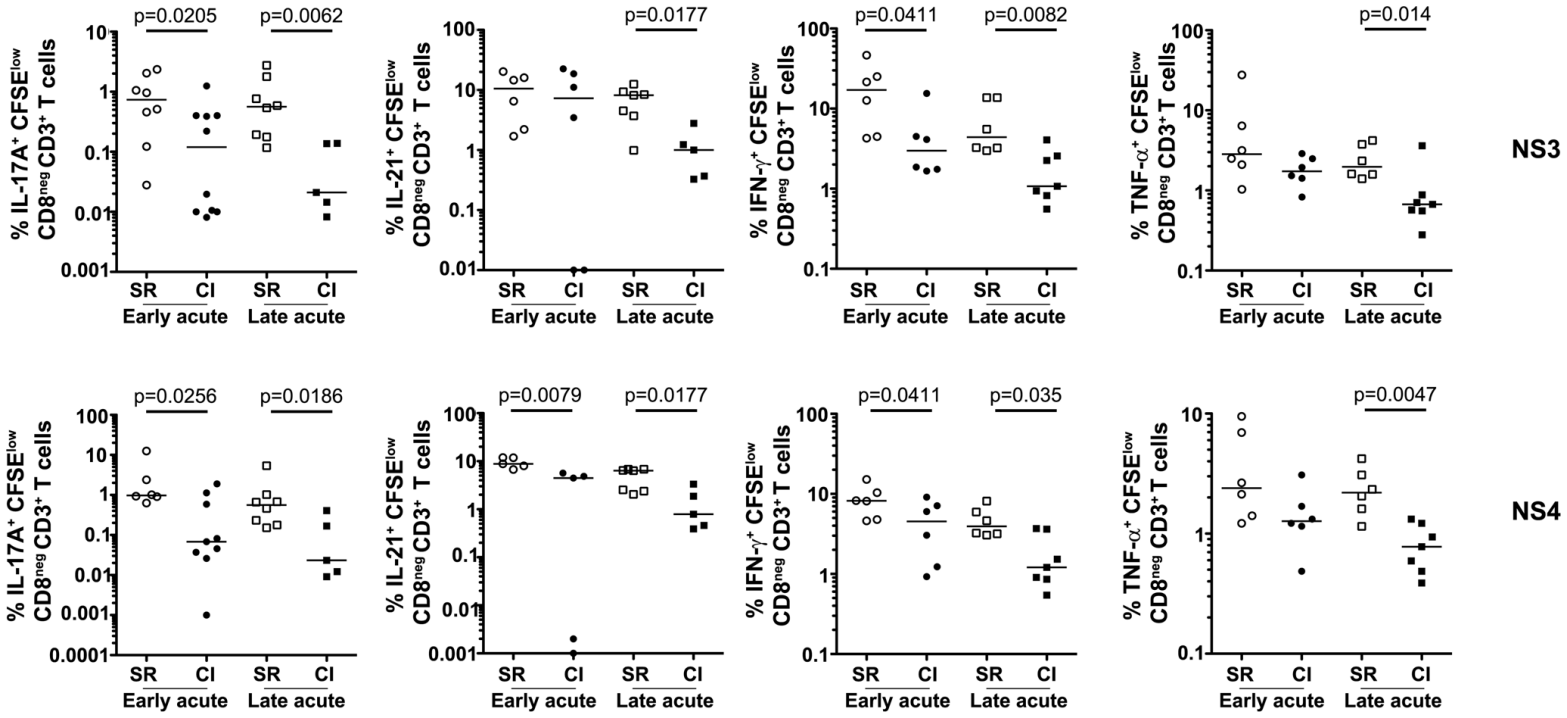
Increased frequency of NS3 and NS4-specific CD4 T cells producing Th17 and Th1 cytokines during acute resolving HCV. CD25-depleted PBMC from patients with acute HCV infection (SR or CI) were stained with CFSE and stimulated with 1 µg/ml of HCV-recombinant proteins (NS3 or NS4). After 6 days of culture, cells were washed and re-stimulated with PMA/ionomycin as described in Materials and Methods to reveal the cytokine profile by ICS. Cells were gated on viable CD3^+^CD8**^neg^** T cells to define the percent of IL-17A-, IL21-, IFN-γ– or TNF-α–producing CFSE^low^ cells. Data is presented as percent specific cytokine production after subtraction of background corresponding to un-stimulated cells. Samples from SR (n = 8-5) and CI (n = 10-5) patients were studied. Samples were not available for some patients at every time point or exhibited low proliferation. Samples from five SR and five CI patients were examined at all the time points tested. P-values were calculated using a two-tailed Mann Whitney U test.

We used this CFSE/ICS assay to examine the functional profile of HCV-specific CD4 T cells in a cohort of injection drug users (IDUs) during acute HCV infection progressing towards either spontaneous resolution (SR) or chronic infection (CI). Patients were recruited and followed as described in Materials and Methods. A summary of the patients' characteristics and demographics is presented in Table 1. Two time- points were tested: i) early acute phase (10 wks±1 wk post estimated date of infection (PEI)) and ii) late acute phase of infection (30 wks±6 wks PEI). As previously described [4], [33]–[35] NS3- and NS4-specific proliferative responses were significantly higher in SR than CI patients during early acute HCV infection (Supplementary Figure S1C). Examining the cytokine profile of HCV-specific CD4 T cells demonstrated specific expansion of Th17 (IL-17A- and IL-21-producing) and Th1 (IFN-γ- and TNF-α- producing) cells in SR patients as compared to CI patients in response to stimulation with NS3 and NS4 during both early and late acute infection (Figure 1).

**Table 1:** Characteristics and demographics of HCV patients studied.

Patient group	Number	Male Gender (%)	Median Age (range)	Genotype 1/3/ND^a^	Mean estimated time post infection at recruitment (days)
Acute→Spontaneous Resolution (SR)	13	10/13 (77)	29 (20–43)	4/3/6	43.4
Acute→Chronic evolution (CI)	24	21/24 (87)	30 (18–48)	14/9/1	43.2
HCV Resolver (R)	8	7/8 (87)	42 (24–50)	ND^a^	N/A^b^
HCV Chronic (C)	27	22/27 (81)	37 (20–60)	ND^a^	ND^a^

a ND: Not determined.

b N/A: Not applicable.

To validate the Th17 lineage of the IL-17A- and IL-21-producing CD4 T cells, we monitored the overall expansion of Th17 cells defined as CD161^high^CCR6^+^CD26^+^ [36], [37]. We observed a specific increase in the frequency of Th17 cells in SR patients (SR median value = 13.25%, pre-infection median value = 6.18% (p<0.0001)), but not in CI patients or patients with long-term chronic HCV (CI median value = 6.315%, chronic HCV median value = 4.89% (p = 0.0006)) (Supplementary Figure S3A). We then sorted CD161^high^CCR6^+^CD26^+^ CD4 T cells from the peripheral blood of long-term HCV spontaneous resolvers or chronic patients (Supplementary Figure S3B) and the examined the expression of the Th17 lineage-specific transcription factors RORc and c-MAF (Supplementary Figure S3C). RORc was highly expressed in CD161^high^CCR6^+^CD26^+^ CD4 T cells as compared to the CD161^neg^ CD4 T cells thus validating their Th17 lineage. However, c-MAF mRNA was specifically up-regulated in Th17 cells from resolvers (Supplementary Figure S3C). CD161^high^CCR6^+^CD26^+^ CD4 T cells expressed significantly higher levels of IL-17A and IL-21 as compared to CD161^neg^ CD4 T cells upon stimulation with PMA/ionomycin (data not shown). Altogether, these results confirm the specific expansion of IL-17A^+^IL-21^+^ Th17 CD4 T cells during acute resolving HCV. This is the first description of HCV-specific Th17 cells during acute HCV infection and suggests that these cells and the cytokines they produce may play an important role in spontaneous clearance of HCV.

### Specific increase in plasma levels of IL-21 during the late acute phase in acute resolving HCV

**Figure 2:**
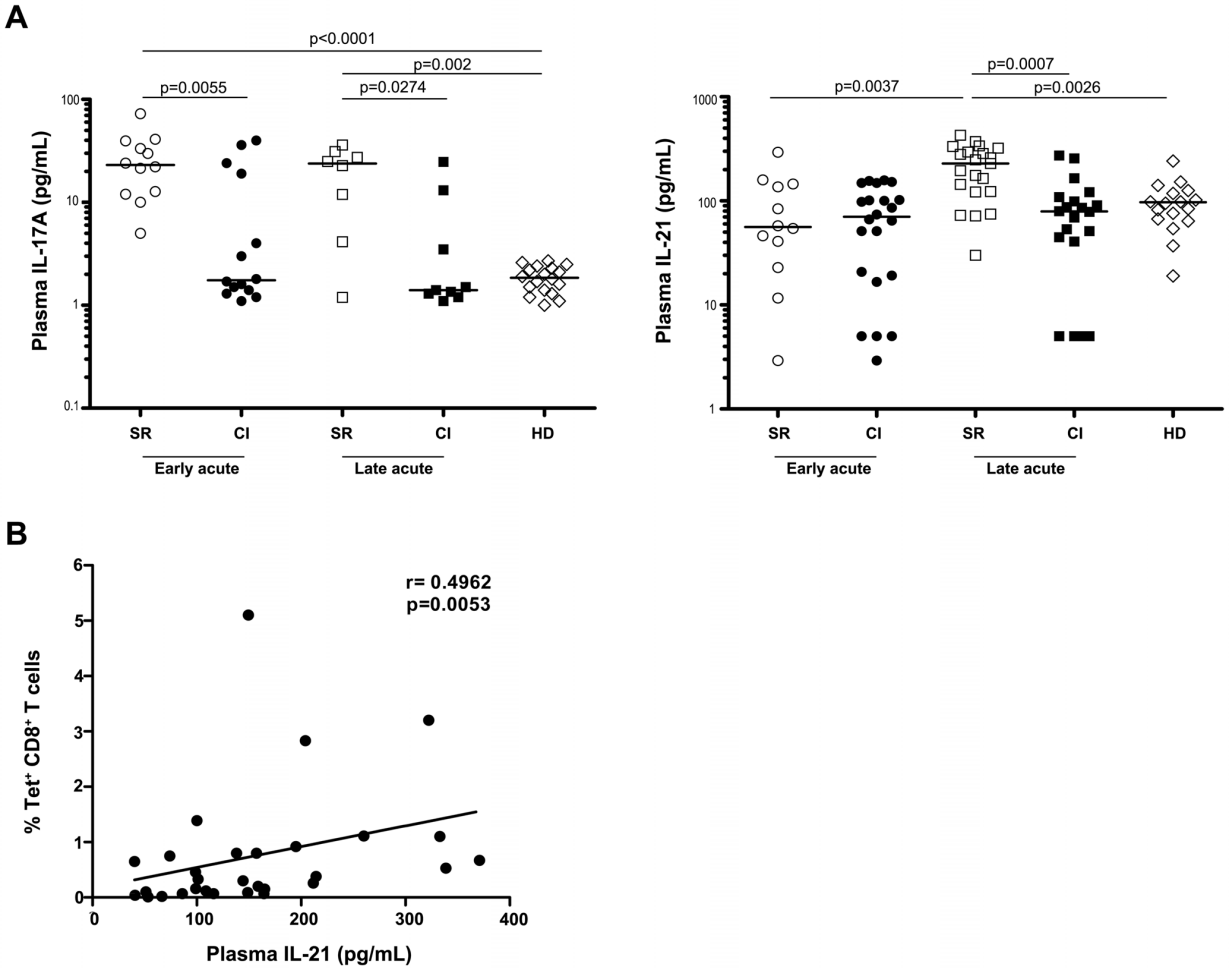
Plasma levels of IL-21 correlate with frequency of HCV-specific CD8 T cells. (**A**) Increased plasma levels of IL-21 during acute resolving HCV. Plasma levels of the Th17 cytokines IL-17A and IL-21 were measured by ELISA at the indicated time points in SR (n = 8–12) and CI (n = 8–22) patients or healthy donors (HD) (n = 17). (**B**) Frequency of HCV-specific CD8 T cells correlates with plasma IL-21 levels. The frequency of HCV-specific CD8 T cells was measured using the following HLA class I tetramers (A1/NS3-1436, A2/NS3-1073, A2/NS3-1406, A2/NS5b-2594, B7/Core-41, B8/NS3-1395 and B27/NS5b-2841) and IL-21 levels were measured in paired plasma samples from 28 acute HCV patients. Correlation was tested using the Spearman correlation test.

In order to assess the role of Th17 cytokines during acute HCV infection, we monitored levels of IL-17A and IL-21 in the plasma of acute HCV patients. SR patients (n = 12) were characterized by an early Th17 response (Figure 2A). Plasma levels of IL-17A were significantly higher at the early acute time point (median value = 23.1 pg/ml) in comparison to healthy donors (HD) (n = 18, p = 0.0001), or to CI patients (n = 14, p = 0.0055). Plasma IL-17A levels remained elevated in SR patients during the late acute phase of HCV infection (median value = 23.9 pg/ml, n = 8), as compared to healthy donors (p = 0.002) and to CI patients (p = 0.0274, n = 9). IL-21 displayed a different pattern of expression. Plasma levels were comparable in SR (n = 12) and CI patients (n = 22) during the early acute phase but increased significantly during the late acute phase in SR patients (median value = 212 pg/ml, n = 21) as compared to CI patients (median value = 79.1 pg/ml, p = 0.0007, n = 19) and healthy donors (median value = 95 pg/m, p = 0.0026, n = 17) (Figure 2A). This is consistent with results in the literature suggesting that IL-21 is more crucial during the memory than the primary phase of antiviral responses [27]–[29].

### Plasma IL-21 levels correlate with the frequency of HCV-specific CD8 T cells

IL-21 can exert its helper effect through enhancing survival and proliferation of virus specific-CD8 T cells and preventing their exhaustion and/or apoptosis by persistent antigenic stimulation. Hence, we examined if the enhanced production of IL-21 during acute HCV influences the frequency of HCV-specific CD8 T cells. Indeed, we could establish a positive correlation between the plasma levels of IL-21 and the frequency of HCV-specific CD8 T cells detected by a panel of 7 MHC class I tetramers (n = 28; r = 0.49 and p = 0.0053) (Figure 2B). These results suggest that IL-21 provides some form of help in the maintenance of virus-specific CD8 T cell responses during acute HCV.

### Different patterns of HCV-specific CD8 T cell exhaustion

**Figure 3:**
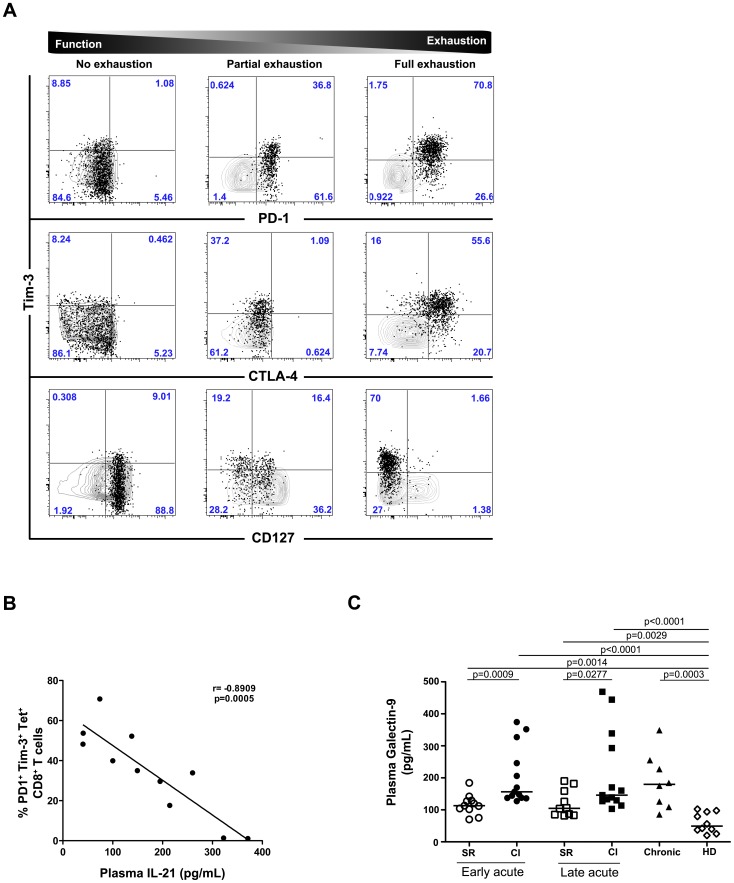
Exhaustion of HCV-specific CD8 T cells and increased Galectin-9 levels during acute HCV with chronic evolution. (**A**) Level of expression of Tim-3 defines differential exhaustion statuses of HCV-specific CD8 T cells during acute infection. Expression of PD-1, CD127, CTLA-4 and Tim-3 on HCV tetramer^+^ (dot plot) or total CD8 T cells (contour plot) in SR (at day 22, left) or in CI patients (at days 62 and 51, middle and right, respectively). Data are representative of 11 different patients analyzed corresponding to all 3 categories. (**B**) Exhaustion of HCV-specific CD8 T cells correlates negatively with plasma IL-21 levels. The co-expression of PD-1 and Tim-3 at the surface of HCV-specific CD8 T cells was measured using the panel of 7 HCV HLA class I tetramers and IL-21 levels were measured in paired plasma samples from 11 acute HCV patients as described in Materials and Methods and Figure 2. Correlation was tested using the Spearman correlation test. (**C**) Increased Galectin-9 levels in plasma during acute HCV infection with chronic evolution. Gal-9 was quantified in plasma by ELISA at the indicated time points during acute HCV infection in SR (n = 11) and CI (n = 13) patients, long-term chronic HCV patients (n = 8) or healthy donors (HD) (n = 10). P-values were calculated using a two-tailed Mann Whitney U test.

Several studies have previously demonstrated that HCV-specific CD8 T cells up-regulate the expression of different exhaustion markers including PD-1, Tim-3 and CTLA-4 during persistent viral replication and progression towards chronic infection. Expression of such markers and their different combinations were associated with different levels of functional impairment in the antiviral properties of HCV-specific CD8 T cells [16], [38], [39]. To evaluate the role of IL-21 in modulating T cell exhaustion, we first sought to determine the combined expression profile of the exhaustion markers PD-1, Tim-3, CTLA-4 and the memory marker CD127 on virus-specific CD8 T cells directly *ex vivo* during acute HCV (n = 9). Based on the levels of Tim-3 expression we were able to define three distinct patterns: Tim-3^neg^ for no exhaustion Tim-3^low^ for partial exhaustion and Tim-3^high^ for a fully exhausted phenotype. Tim-3^neg^ cells were mostly detected in SR patients (n = 4) and were found to be negative for the exhaustion markers PD-1 and CTLA-4 but expressed high levels of the memory marker CD127, associated with poly-functional T cells [40]. In contrast, Tim-3^low^ and Tim-3^high^ cells were detected in CI patients during acute HCV (n = 7). Tim-3^low^ cells were PD-1^high^CTLA-4^neg^ and expressed intermediate levels of CD127. Tim-3^high^ CD8 T cells were PD-1^high^CTLA-4^high^ but CD127^neg^ (Figure 3A). These diverse expression patterns suggest different exhaustion statuses based on the increased expression of Tim-3, PD-1 and CTLA-4 and the reduced expression of CD127. Indeed, we observed a reduction in the proliferative capacity of HCV-specific cells in response to cognate HCV peptides with increased expression of Tim-3 (Supplementary Figure S4). Given that the frequency of HCV-specific T cells correlated with plasma levels of IL-21, we hypothesized that IL-21 may preserve the functions and limit the exhaustion of HCV-specific T cells and enhance their survival. We examined the correlation between the frequency of exhausted T cells (PD-1^+^Tim-3^+^HCV-tetramer^+^ CD8 T cells) and the plasma levels of IL-21. We established a negative correlation between these two parameters (Figure 3B, p = 0.0005, n = 11). This suggests that the frequency and the functionality of HCV-specific CD8 T cells may be dependent on IL-21, a T-helper cytokine.

### Increased plasma levels of the Tim-3 ligand Galectin-9 during acute HCV

Tim-3 has recently emerged as a major determinant of the functional status of HCV-specific T cells [16], [39]. The inhibitory effect of Tim-3 during the majority of chronic viral infections is dependent on interaction with its main ligand Gal-9 [15], [20], [41], [42]. Consequently, we decided to quantify the levels of Gal-9 in plasma during acute HCV infections with different outcomes. Gal-9 was specifically higher in CI (n = 13) as compared to SR (n = 11) patients during the early and late acute phases of HCV (p = 0.0009 and p = 0.0277, respectively) or as compared to healthy donors (n = 10, p<0.0001). Gal-9 was also significantly elevated in the plasma of long-term HCV chronic patients (n = 8) as compared to healthy donors (p = 0.0003) (Figure 3C).

### Expansion of Galectin-9-expressing CD39^+^ Tregs during acute HCV with chronic evolution

**Figure 4:**
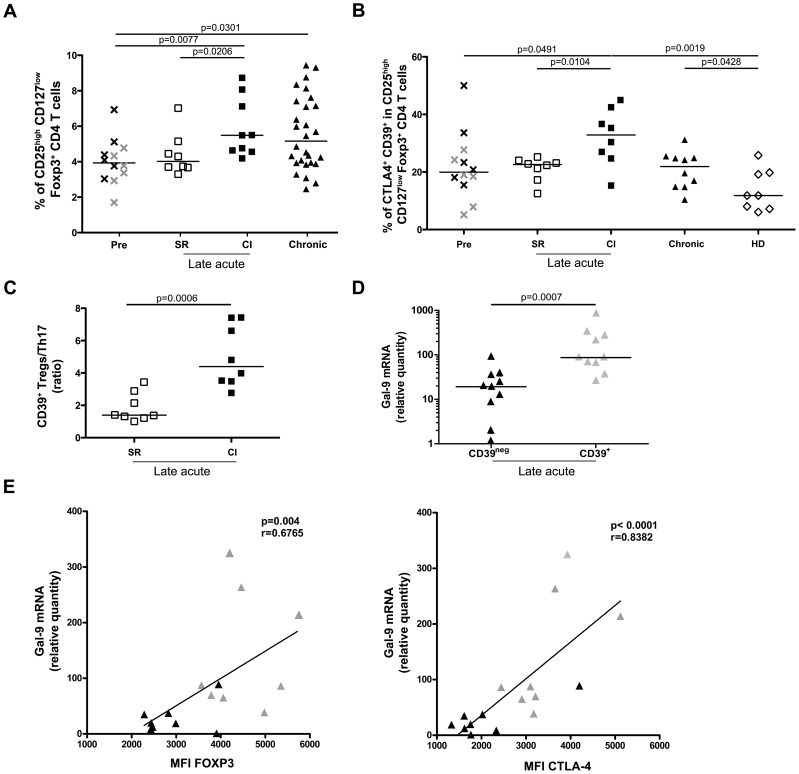
Expansion of Gal-9^+^ Tregs during acute HCV infection with chronic evolution. (**A**) Increased frequency of Tregs in CI patients during late acute HCV. Regulatory T cells were identified *ex vivo* as CD127^low^CD25^high^FoxP3^+^ CD4 T cells in PBMCs from HCV patients at pre-infection (n = 12, grey symbols represent SR and black symbols represent CI), late acute (n = 8 for SR, n = 9 for CI) and long-term chronic HCV patients (n = 22). P-values were calculated using a two-tailed Mann Whitney U test. (**B**) Increased frequency of CD39^+^ CTLA-4^+^ Tregs in CI patients during late acute HCV. Tregs were defined as CD127^low^CD25^high^FoxP3^+^ CD4 T cells in pre-infection samples (n = 12, grey symbols represent SR and black symbols represent CI), late acute phase in SR (n = 8) and CI (n = 8) patients, long-term HCV chronic patients (n = 10) and healthy donors (n = 8). P-values were calculated using a two-tailed Mann Whitney U test. (**C**) Imbalance between CD39^+^CTLA-4^+^ Tregs and Th17 cells during acute HCV infection. The ratio between the frequencies of CD39^+^CTLA-4^+^ Tregs and CD161^high^CCR6^+^CD26^+^ CD4 T cells was calculated during late acute HCV infection in SR and CI patients (n = 8). P-values were calculated using a two-tailed Mann Whitney U test. (**D**) CD39^+^ Tregs express Galectin-9 in HCV infected patients. Gal-9 mRNA expression in CD39^+^ (grey triangles) and CD39^neg^ (black triangles) FACS-sorted Tregs from HCV patients was measured by real-time PCR and normalized to 18S mRNA expression. Relative expression was calculated relative to the expression of *LGALS9* in FACS-sorted CD127^high^ CD25^neg^ CD4 T cells (purity>98%). Gal-9 mRNA expression was higher in CD39^+^ than CD39^neg^ Tregs (p<0.01) (n = 10). P-values were calculated using a two-tailed Mann Whitney U test. (**E**) CD39^+^ CTLA-4^+^ Tregs express the highest levels of Galectin-9. CD39^+^ (grey triangle) and CD39^neg^ (black triangle) Tregs were sorted from chronic HCV patients (n = 8) and stained for expression of FoxP3 and CTLA-4. Gal-9 mRNA expression in sorted cells was evaluated by real-time PCR and normalized to 18S mRNA expression. Relative expression was calculated relative to expression of *LGALS9* in FACS-sorted CD127^high^ CD25^neg^ CD4 T cells (purity>98%). Correlation with FoxP3 and CTLA-4 expression was performed using Spearman correlation test.

To further dissect the mechanisms contributing to the loss of the helper T cell response and, consequently, the inhibition of the CD8 T cell antiviral function, we examined the specific emergence of suppressive Tregs during acute HCV infections progressing to chronicity. First, we examined the frequency of peripheral CD4 Tregs using the classical markers CD25^high^ CD127^low^ and FoxP3^+^. We observed a higher frequency of Tregs in CI patients during the late acute phase of HCV (n = 9) as compared to pre-infection (p = 0.0077, n = 12), SR (p = 0.0206, n = 8) and long-term chronic HCV patients (p = 0.0301, n = 28) (Figure 4A). In depth phenotypic characterization demonstrated an increase in the frequency of Tregs co-expressing CTLA-4 and the ecto-enzyme CD39 in CI patients during the late acute phase of HCV infection (p = 0.0491 *vs* pre-infection; p = 0.0104 *vs* SR and p = 0.0019 *vs* HD) (Figure 4B). As previously described, this subset of Tregs possesses a high suppressive potential, notably the inhibition of Th17 cells [43]. Thus, we sought to determine if the Treg/Th17 ratio is altered during acute HCV infection as observed during simian immunodeficiency virus (SIV) infection [44]. An imbalance in the ratio of CD39^+^ CTLA-4^+^ Tregs and of CD161^high^CCR6^+^CD26^+^ CD4 T cells was observed during late acute HCV infection (Figure 4C, p = 0.0006, n = 8 for SR and CI patients).

Recent data in HIV patients has demonstrated that Tregs can be a source of Gal-9 production [20]. Given the expansion of CD39^+^ Tregs and the increase in plasma levels of Gal-9 in CI patients, we hypothesized that this subset may be a source of Gal-9 in CI patients. To test our hypothesis, CD39^+^ and CD39^neg^ Tregs from chronic HCV patients were sorted by FACS. The quantification of gene expression by real-time PCR demonstrated that CD39^+^ Tregs (grey symbols) expressed higher levels of Gal-9 than CD39^neg^ Tregs (black symbols) in chronic HCV patients (p = 0.0007, n = 10) (Figure 4D). Furthermore, we established a positive correlation between the expression of Gal-9 and the mean fluorescence intensity (MFI) of FoxP3 (p = 0.004) and CTLA-4 (p<0.0001) (Figure 4E). In summary, we observed a strong association between expression of CTLA-4, CD39 and Gal-9 in FoxP3^+^ Tregs. This was coupled with the expansion of this T cell population during late acute HCV with a chronic evolution and the temporal decrease in HCV-specific Th17 cells in the same patients. These two observations suggest a fine balance between inflammatory and regulatory cells that may influence the outcome of the infection.

### Exhaustion as a potential mechanism for failure of IL-21-producing HCV-specific CD4 helper T cells

**Figure 5:**
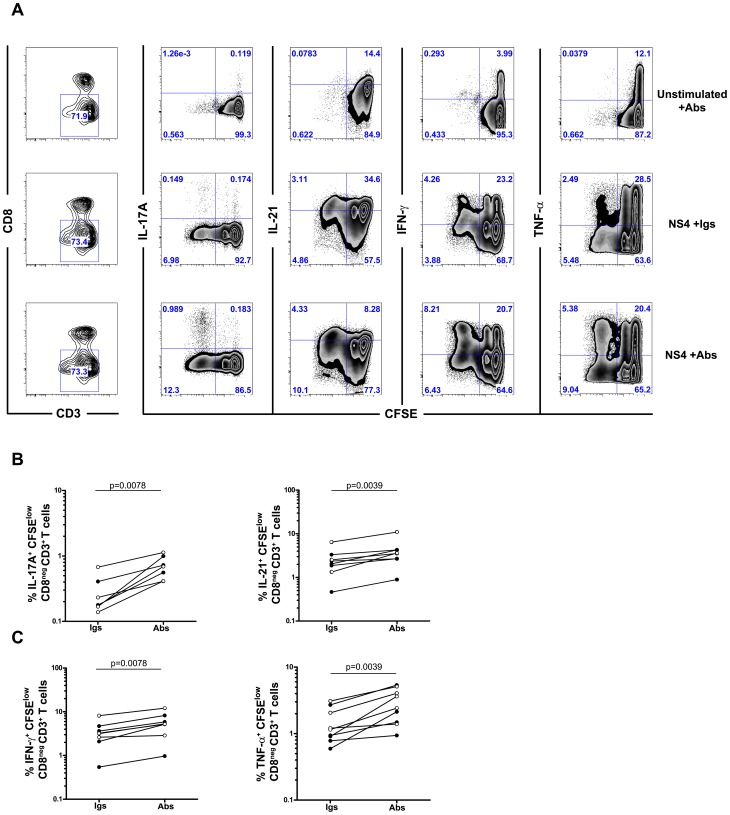
Restoration of HCV-specific IL-17A and IL-21 production by blocking multiple inhibitory pathways. (**A**) Representative FACS plot demonstrating enhanced production of Th17 and Th1 cytokines by HCV NS4-specific CD4 T cells upon antibody blockade. CD25^neg^ PBMCs from acute HCV patients were stained with CFSE and stimulated with 1 µg/ml of HCV NS4 recombinant protein in the presence of control Igs (IgG1, Ig2a) or blocking antibodies against PD-L1, CTLA-4 and Tim-3 (10 µg/ml each). After 6 days of culture, cells were washed and re-stimulated with PMA/ionomycin to detect production of Th17 (IL17A, IL-21) and Th1 (IFN-γ and TNF-α) cytokines by ICS as described in Materials and Methods. Cells are gated on CD3+CD8^neg^ lymphocytes (left panels). (**B, C**) Enhanced cytokine expression upon antibody blockade in the SR (open symbols, n = 3–5) and CI patients (closed symbols, n = 3–5). Data is represented as percent cytokine expressing CFSE^low^ cells within the CD3^+^CD8^neg^ lymphocyte gate. P-values were calculated using Wilcoxon signed rank test.

We have demonstrated that progression to chronic HCV infection is associated with a shift from Th1/Th17 virus-specific CD4 T cells to Gal-9-expressing Tregs. This led to a failure in the maintenance of CD4 T cells which produce a key helper cytokine, IL-21. This prompted us to investigate if the reduced frequency of IL-21 producing Th17 cells and therefore, the reduced levels of IL-21 in the plasma could be the mechanism restricting CD4 T cell help in acute infections with chronic evolution. IL-21 production could be limited directly through exhaustion of IL-21 producing Th17 cells or indirectly through modulation of Th17 cell function and cytokine producing capacity by inhibitory Tregs. Given that the exhaustion of HCV-specific Th17 cells could not be evaluated directly *ex vivo* because of a lack of appropriate HCV MHC class II tetramers, we investigated whether IL-17A and/or IL-21 could be restored upon the blockade of several inhibitory pathways in 6 patients during the late acute phase of HCV infection (30 wks±6 wks PEI). As described above, we used the combined CFSE/ICS assay to monitor the restoration in proliferation and to determine the functional profile of HCV-specific CD4 T cells following stimulation with the HCV NS4 antigen in the presence of isotype control antibodies or of neutralizing antibodies directed against PD-L1, CTLA-4 and Tim-3. We used this triad of blocking molecules to optimize the functional restoration of exhausted T cells. At the end of the 6 days CFSE proliferation assay, cells were stimulated with PMA/ionomycin to assess the intracellular production of cytokines. The restoration in cytokine production upon blockade of the inhibitory pathways is depicted in Figure 5A. Blocking the inhibitory pathways slightly enhanced the proliferation of HCV-specific CD4 T cells in response to NS3 and NS4 (data not shown). Nevertheless, this blockade led to a significant increase in the percent of NS4-specific proliferating T cells which produce the Th17 cytokines, IL-17A and IL-21 (p = 0.0078 and p = 0.0039, respectively) (Figure 5B) and the Th1 cytokines, IFN-γ and TNF-α (p = 0.0078 and p = 0.0039, respectively) (Figure 5C). Similar results were observed upon NS3 stimulation (data not shown). Restoration was also tested in an additional group of 6 patients who had HCV-specific CD4 responses during the early acute phase that became undetectable during the late acute phase. Neutralizing the inhibitory pathways did not restore proliferation or cytokine production by CD4 T cells in this group suggesting that immune restoration is dependent on the maintenance of a minimal frequency of HCV-specific T cells (data not shown). Altogether, these data suggest that HCV-specific Th17 cells are exhausted during acute HCV with chronic evolution and that there is a narrow window of opportunity where exhaustion can be reversed via interference with the implicated inhibitory pathway(s).

### IL-21 rescues proliferation of exhausted HCV-specific CD8 T cells

**Figure 6:**
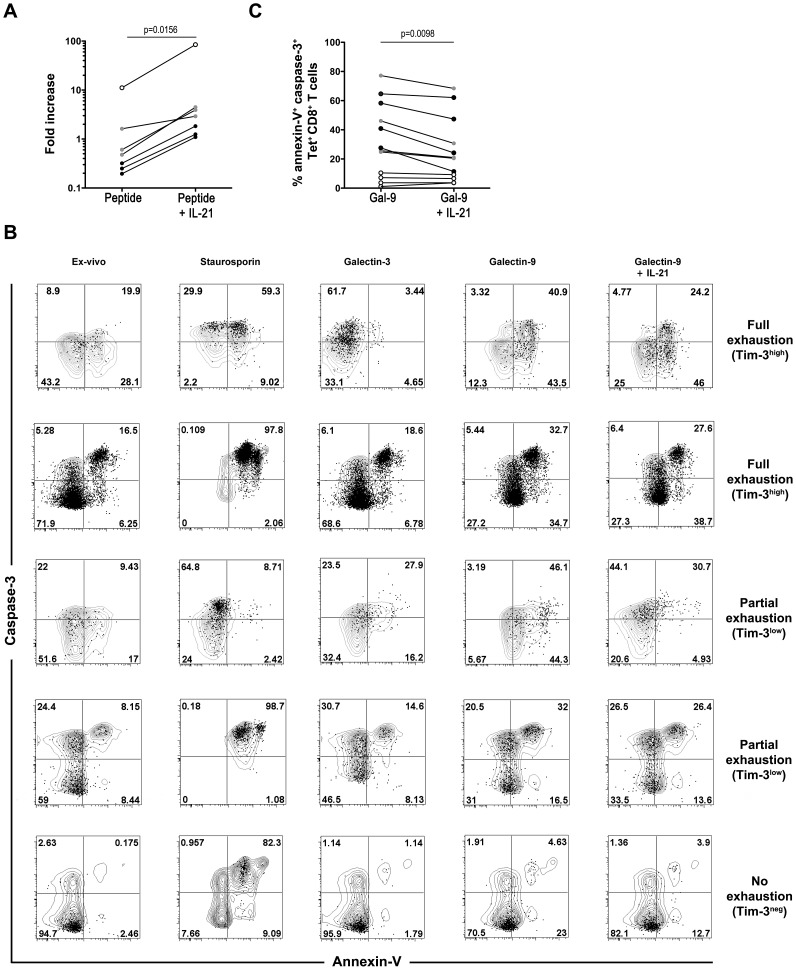
IL-21 enhances proliferation and limits Galectin-9 induced apoptosis in Tim-3^high^ HCV-specific CD8 T cells. (**A**) IL-21 enhances expansion of HCV-specific CD8 T cells in a Tim-3-dependent manner. PBMCs from acute HCV patients were stimulated with their cognate peptide epitopes corresponding to the HCV MHC class I tetramers used, in the presence or absence of IL-21. Patients were classified according to Tim-3 expression on HCV tetramer^+^ CD8^+^ T cells as: Tim-3^neg^ (open circles), Tim-3^low^ (grey circles) and Tim-3^high^ (closed circles). Fold expansion was calculated by dividing the frequency of HCV tetramer^+^ CD8^+^ T cells after *in vitro* expansion by the frequency measured directly *ex vivo*. P-values were calculated using Wilcoxon signed rank test. (**B**) IL-21 counteracts Galectin-9-induced apoptosis. PBMCs were incubated for 4 hours in the presence of Staurosporin (1 µM), a protein kinase C (PKC) inhibitor (used as a positive control for induction of apoptosis), Gal-3 (negative control) or Gal-9 (1 µM), in the presence or absence of IL-21. Cells were then stained with HCV tetramers and antibodies against cell surface markers. Apoptosis was measured by surface staining with Annexin V followed by fixation and permeabilization, before intracellular staining for caspase-3. Representative data of PBMCs from patients during acute HCV expressing high (top), low (middle) or negative (bottom) levels of Tim-3 on the surface of HCV-tetramer+ CD8 T cells and corresponding to different levels of exhaustion as described in Figure 3 (n = 2 for full exhaustion, n = 2 for partial exhaustion, and n = 1 for no exhaustion). Patients tested were all in the early acute phase (day 22–124). (**C**) IL-21 counteracts Galectin-9-induced apoptosis of HCV-specific CD8 T cells. Gal-9-induced apoptosis in the presence or absence of IL-21 was measured by the co-expression of caspase-3 and Annexin V on HCV-specific tetramer+ CD8 T cells expressing different levels of Tim-3 during early acute HCV infection (day 22–124) (n = 12) as described in Figure 6B. Tim-3^neg^ (open circles), Tim-3^low^ (grey circles) and Tim-3^high^ (closed circles). P-values were calculated using Wilcoxon signed rank test.

To confirm the key helper role of IL-21 during acute HCV, we investigated whether exogenous supplementation of IL-21 can rescue the proliferative capacity of exhausted HCV-specific T cells. The addition of IL-21 to a 6 day proliferation assay stimulated with the cognate HCV peptide led to a significant expansion of tetramer-positive cells in all patient samples collected at the early acute time point (n = 7; p = 0.0379). Although IL-21 enhanced proliferation of all HCV-specific CD8 T cells, its effect was more prominent on Tim-3^low^ and Tim-3^high^ cells (Figure 6A).

### IL-21 rescues HCV-specific CD8 T cells from Galectin-9 induced apoptosis

We have demonstrated that HCV-specific CD8 T cells can develop different levels of exhaustion according to the level of expression of Tim-3. Furthermore, we demonstrated that progression to chronic HCV-infection is associated with lower plasma levels of IL-21, increased Gal-9 and increased frequencies of Gal-9 expressing Tregs. Gal-9 can induce apoptosis of T cells upon interaction with its ligand Tim-3 [41] and may thus contribute to the inhibition of Tim-3^+^ HCV-specific CD8 T cells. So, we hypothesized that IL-21 may rescue HCV-specific cells from Gal-9-induced apoptosis. The anti-apoptotic effect of IL-21 was tested during a short-term exposure of HCV-specific T cells expressing different levels of Tim-3 to Gal-9. PBMCs collected from SR or CI patients during acute HCV (n = 12) were stimulated with Gal-9; the irrelevant ligand Galectin-3 (Gal-3) (used as a negative control) or Staurosporin, an inhibitor of protein kinase C (PKC) (used as a positive control). Apoptosis was assessed by examining the co-expression of Annexin-V and caspase 3 in total CD8 and HCV-tetramer^+^ T cells. Representative data of the responses from 5 patients with different patterns of Tim-3 expression (Tim-3^high^ (n = 2), Tim-3^low^ (n = 2) and Tim-3^neg^ (n = 1)) are shown in Figure 6B. Spontaneous apoptosis was higher directly *ex vivo* in Tim-3^high^ cells as compared to Tim-3^low^ and Tim-3^neg^ cells. Furthermore, the addition of Gal-9 but not Gal-3 triggered apoptosis in 1.13 to 77.2% (median value 26.6%) of HCV tetramer^+^ CD8 T cells from all HCV patients. Cell death was found to be more pronounced in patients with higher levels of T cell exhaustion (Figure 6B). The addition of IL-21 reduced this Tim-3 mediated apoptosis to 3.66 to 68.4% (median value 20.75%) (p = 0.0098), which is similar to the non-specific apoptosis induced by Gal-3 (Figure 6C). This data suggest that IL-21 may enhance the survival of HCV-specific CD8 T cells *in vivo* by down-modulation of the Gal-9/Tim-3-induced apoptosis.

### Knockdown of Galectin-9 reduces Treg-mediated inhibition of IL-21 production

**Figure 7:**
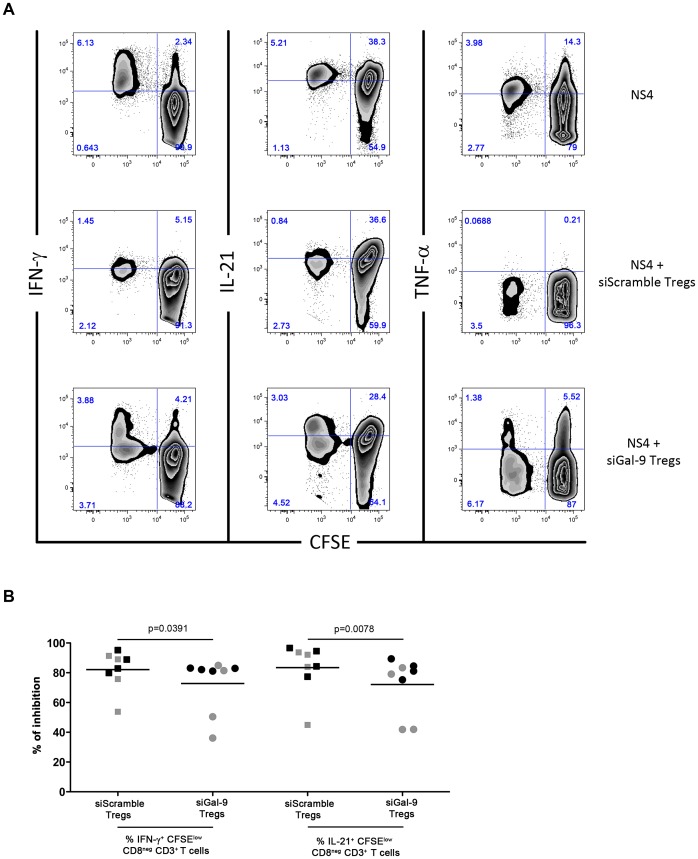
Galectin-9 knockdown limits Treg-mediated inhibition of IFN-γ and IL-21 production by HCV-specific CD4 T cells. (**A**) Representative combined CFSE/ICS assay demonstrating reduced Treg-mediated inhibition upon knockdown of Gal-9 by siRNA. Plots depict production of IFN-γ, IL-21 and TNF-α by HCV-specific proliferating (CFSE^low^) T cells in response to stimulation with recombinant HCV NS4 protein. PBMC cultures depleted of Tregs (Top panel) or with addition of Tregs (ratio 1∶4, Tregs∶CD25-depleted PBMCs) treated with either control siRNA (Middle panel) or *LGALS9* siRNA were stimulated as described in Materials and Methods. Plots are gated on CD3^+^ CD8^neg^ lymphocytes. (**B**) Percentage of Treg-mediated inhibition of IFN-γ and IL-21 production by HCV-specific T cells upon stimulation with recombinant HCV proteins (NS3: black symbols or NS4: grey symbols) in the presence of Treg cells treated with either control or *LGALS9* siRNA, as described in (A) and Materials and Methods. The percentage inhibition of cytokine production upon addition of Tregs to the stimulated PBMC cultures was calculated using the following formula: (% CFSE^low^cytokine^+^CD8^neg^ T cells in absence of Tregs – % CFSE^low^cytokine^+^CD8^neg^ T cells in presence of Tregs)/% CFSE^low^cytokine^+^CD8^neg^ T cells in absence of Tregs×100. P-values were calculated using Wilcoxon signed rank test.

We have demonstrated the implication of the Gal-9/Tim-3 pathway in the functional inhibition of both HCV-specific CD4 and CD8 T cells and that IL-21 may reverse this inhibitory effect. In addition, we have demonstrated the expansion of Gal-9-expressing Tregs during acute HCV with chronic evolution. Thus, we hypothesized that Tregs may contribute to inhibiting HCV-specific IL-21 production not only through the production of the classical Treg immuno-modulatory cytokines like TGF-β or IL-10, but also through the activation of the Gal-9/Tim-3 inhibitory pathway. In order to investigate this hypothesis, we performed a combined CFSE/ICS in the presence of regulatory T cells transfected with siRNA-specific for the Gal-9 gene (*LGALS9*) or control siRNA (scramble sequence) (Supplementary Figure S5A). The efficiency of gene knockdown was evaluated by quantitative RT-PCR (qRT-PCR) after transfection with specific or scrambled siRNA (Supplementary Figure S5B). Tregs were added to the cultures at a ratio of 1∶4 (Tregs∶CD25-depleted PBMCs) as described by Elahi et al [20]. The addition of Tregs transfected with scrambled siRNA to cultures inhibited the proliferation of HCV-specific T cells in the samples from CI patients collected during the early acute phase. The production of IFN-γ and IL-21 by HCV-specific CD4 T cells (CD3^+^CD8^neg^) was also reduced by nearly 80% in comparison to cultures without Tregs (Figure 7A, B). In contrast, Tregs transfected with siRNA specific to *LGALS9* reduced the Treg-mediated inhibition of HCV-specific CD4 T cells secreting IFN-γ and IL-21 (p = 0.0391 and p = 0.0078 respectively). The frequencies of HCV-specific IL-17A- and TNF-α- producing CD4 T cells, were not altered by the silencing of Gal-9. However, the number of virus-specific Th17 cells was too low in these CI patients to establish definitive conclusions.

Altogether, these data suggest that the proliferation of HCV-specific T cells secreting IL-21 may be partially controlled by Gal-9^+^CD39^+^ Tregs during acute HCV infection in CI patients. The delicate balance in frequencies between this cell subset and IL-21-producing Th17 cells may be one of the determinants of the outcome of acute HCV.

## Discussion

In this study, we demonstrated an important role for IL-21 as a major helper cytokine during acute HCV infection by limiting the T cell dysfunction induced by the Gal-9/Tim-3 interaction. We have demonstrated a direct correlation between higher levels of IL-21 production and cell proliferation, as well as cell survival and the inhibition of exhaustion of HCV-specific CD8 T cells. Moreover, our results have identified three different mechanisms of CD4 helper T cell failure during acute HCV infections with chronic evolution. First, the exhaustion of HCV-specific helper T cells may lead to decreased IL-21 production and failure to sustain efficient CTL responses. Second, an imbalance between inflammatory (Th17) and regulatory (Treg) CD4 T cells may have a direct inhibitory effect on HCV-specific CTL responses. Third, we have identified CD39^+^ Tregs as a potential source of Gal-9 during chronic HCV infection and demonstrated that Gal-9-expressing Tregs can directly inhibit proliferation and IL-21 production by HCV-specific CD4 T cells. These mechanisms combined may limit CD4 T cell help, trigger exhaustion and apoptosis of HCV-specific T cells and favor virus persistence.

The helper role of IL-21 during acute HCV can be mediated through direct and indirect effects. IL-21 acts directly to prevent exhaustion [27]–[29], and to enhance the cytotoxic capacity of virus-specific CTLs through the up-regulation of perforin [45]–[47] and granulysin [48]. Also, it sustains the proliferation and survival of virus-specific memory CTLs [49] and decreases senescence and susceptibility to apoptosis through the modulation of caspase-3 expression [50]. Indirectly, IL-21 production by Th17 cells favors their development by increasing the expression of the IL-23 receptor (IL-23R) and thus enhancing the sensitivity to this Th17-polarizing cytokine [51], [52]. IL-21 also inhibits the differentiation of Tregs by interfering with FoxP3 expression [53]–[55]. Moreover, it can counteract Treg-mediated suppression by inhibiting T cell IL-2 production, which leads to the impairment of Treg homeostasis through IL-2 deprivation [56].

We have observed a specific increase in the frequency of IL-21-producing Th17 cells, identified as CD161^high^CCR6^+^CD26^+^ CD4 T cells, during acute resolving HCV. The limited expansion of this subset seen in acute infections with chronic evolution can be due to its exhaustion status. Indeed, we have demonstrated that blocking the PD-1/Tim-3 and CTLA-4 inhibitory pathways can rescue IL-21 production. Another possibility is the modulation of the inhibitory effect of Tim-3 via human leukocyte antigen B (HLA-B)-associated transcript 3 (Bat3) [57]. We have observed lower expressions of Bat3 in Th17 cells from chronically infected HCV patients when compared to SR patients (data not shown). This is similar to features of T cell exhaustion during chronic HIV infection as well as in a mouse model of cancer [57].

IL-21-producing Th17 cells may preferentially home to the liver. Several studies have reported an increase in the frequency of Th17 cells in the livers of patients with chronic liver diseases, including infections with HBV and HCV [23], [58], [59]. Furthermore, the expression of CD161 on liver-resident HCV-specific IL-17-producing CD8 T cells (Tc17 cells) was found to be tightly linked to the expression of CXCR6, a liver homing chemokine receptor [60]. Due to ethical constraints that restrict any liver biopsies during acute HCV infection, we examined the homing potential of Th17 cells from SR and CI patients by evaluating the expression of the homing receptors CCR5, CXCR3 and CXCR6. However, we did not observe any significant differences between the two groups (data not shown). This result does not exclude the possibility that such liver homing cells were not detected because they are no longer in circulation. Similarly, we could not assess the contribution of other IL-21-producing cellular populations, such as follicular helper T cells which are mainly found in the lymph nodes and NKT cells which are mainly found in the liver.

We have observed three levels of expression of the inhibitory receptor Tim-3 on HCV-specific CD8 T cells. The combination of Tim-3 with PD-1, CTLA-4 and CD127 identified different degrees of functional impairment: un-exhausted (Tim-3^neg^PD-1^neg^CTLA-4^neg^CD127^high^), partially exhausted (Tim-3^low^PD-1^high^CTLA-4^dim^CD127^dim^) and fully exhausted (Tim-3^high^PD-1^high^CTLA-4^high^CD127^neg^) HCV-specific CD8 T cells. Such a hierarchical model of exhaustion was proposed to explain the progressive loss of virus-specific CD8 T cell functions in LCMV [61] and HCV infections [10], [38], [39]. Although we did not observe any significant differences in the expression of CD160 (data not shown), other inhibitory receptors such as 2B4, LAG-3 and KLRG1 may also be critical in defining the exhaustion level of HCV-specific T cells [38], [62], [63]. Moreover, the level of expression may also be affected by the host's HLA genotype. Recent data have demonstrated that HIV patients carrying the protective HLA alleles (HLA-B27 and HLA-B57) exhibited limited up-regulation of Tim-3 at the surface of HIV-specific CD8 T cells after cognate epitope stimulation [20]. In contrast, patients carrying the less protective alleles (including HLA-A3) exhibited higher up-regulation of Tim-3 and were more susceptible to functional impairment. Interestingly, HLA-B27 has been associated with a higher rate of spontaneous resolution in acute HCV infection [64], [65] and it is possible that it may play a similar role in limiting T cell exhaustion during acute HCV. Differential up-regulation of inhibitory receptors could be due to variable affinities of the peptide/MHC interactions with the T cell receptors that lead to multiple thresholds of activation.

A blockade of the three inhibitory receptors, PD-1, Tim-3 and CTLA-4, rescued IL-17A and IL-21 production by HCV-specific CD4 T cells. This suggested that these pathways are implicated in helper T cell failure and exhaustion during acute HCV. In addition, it suggested that the hierarchical model of T cell exhaustion, previously observed in CD8 T cells, is also applicable to CD4 T cells. Indeed, gradual and progressive loss of CD4 helper function was observed in HCV infections progressing to chronicity where the earliest function lost was IL-2 production followed by proliferation then IFN-γ production [7]. In the present study, we could not restore CD4 T cell function in patients with chronic evolution where the response was undetectable during the late acute phase suggesting that there is a minimal threshold of T cell frequency and a window of opportunity for immune restoration to succeed. Interestingly, the blocking of all three pathways on exhausted HCV-specific CD8 T cells restored proliferation, but only Tim-3 blockage restored cytotoxic function [39]. Data from HIV and LCMV infections suggest that the level of expression of each inhibitory receptor could also dictate the efficiency of immune restoration upon its blockade [61], [66]. Given the difficulty in phenotyping HCV-specific CD4 T cells directly *ex vivo*, further research is still needed to examine the individual contribution of each inhibitory receptor in defining the level of CD4 T cell exhaustion. It is also noteworthy that higher levels of expression of such inhibitory receptors were observed on HCV-specific CD8 T cells in the liver during chronic HCV infection [11]–[16] and this may limit immune restoration strategies and the immuno-modulatory role of IL-21.

Tregs were expanded during the acute phase of HCV infection in CI patients and may exert a direct inhibitory effect on the function of virus-specific T cells. In addition, we observed a specific expansion of Tregs co-expressing CTLA-4 and CD39. CD39^+^ Tregs were also found to be expanded in HIV [67] and chronic HBV infections [68] and were shown to counteract or inhibit the expansion of Th17 cells during remission of patients with multiple sclerosis [69]. Moreover, a polymorphism in the CD39 gene was recently identified and associated with a slow progression to AIDS in HIV-infected patients [70]. Similar genetic susceptibility may explain the differential induction of CD39^+^ Tregs in HCV-infected patients. Other signals driving cross-regulation between regulatory T cells and IL-21-secreting Th17 CD4 T cells may involve TGF-β [71], [72] and/or Notch signaling [73], [74]. In summary, HCV infection may disrupt the balance between Th17 cells and Tregs and, therefore, tamper inflammation while diminishing the effector functions of HCV-specific T cells thus facilitating virus persistence [75].

We have observed the preferential expression of Gal-9 by CD39^+^ Tregs which contributes to their suppressive capacity [76], [77] through interaction with Tim-3 on the surface of exhausted T cells as recently seen in HIV infection [20]. It is important to note that there are other sources of Gal-9 *in vivo*, specifically Kupffer cells in the liver that may contribute to the increase in apoptosis of liver-resident T cells [15]. We demonstrated a direct link between inhibition of HCV-specific T cell function and the expression of Gal-9 by Tregs during acute HCV infection. A delicate balance between IL-21 and Gal-9 can control survival, exhaustion and apoptosis of HCV-specific T cells and could be a major determinant of infectious outcome.

**Figure 8:**
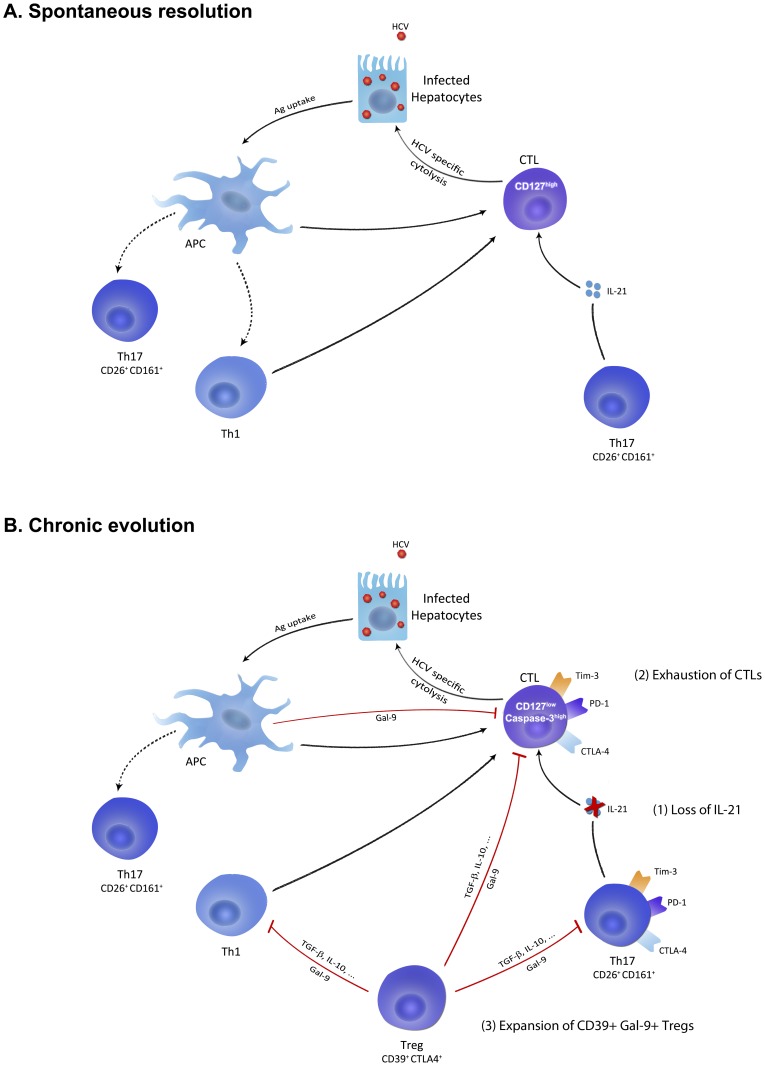
Proposed model for role of Galectin-9 and IL-21 during acute HCV infection. (**A**) HCV infection of hepatocytes and antigen uptake and presentation by liver-resident and circulating antigen presenting cells (APCS) prime HCV-specific CTLs and helper CD4 T cells including Th1 and Th17 cells. Function and survival of HCV-specific CTLs are sustained by increased production of IL-21 by Th17 cells during the late acute phase of spontaneously resolving HCV infection. (**B**) In patients with chronic evolution, multiple inhibitory mechanisms cooperate to induce failure of the immune response and facilitate viral persistence. (1) HCV-specific Th17 cells produce less IL-21 thus compromising CTL survival and function. (2) HCV-specific CTLs become exhausted and up-regulate exhaustion molecules like Tim-3, PD-1 and CTLA-4 and become more susceptible to apoptosis that is aggravated by lack of sufficient IL-21. (3) CD39**^+^** Tregs are increased in frequency and may directly inhibit Th17 cells and Tim-3-expressing CTLs via Gal-9-mediated apoptosis or through classical mechanisms including cellular contact and immuno-modulatory cytokines like IL-10 and TGF-β.

We propose a model where HCV-specific CD4 and CD8 T cells are induced early during acute HCV infection but multiple mechanisms could contribute to failure of the immune response during the late acute phase. First, failure to expand IL-21-producing Th17 cells leads to diminished survival and function of HCV-specific CD8 T cells. Second, failure to control viral replication leads to the exhaustion of both virus-specific CD4 and CD8 T cells due to the up-regulation of different inhibitory receptors including PD-1, Tim-3 and CTLA-4. T cell exhaustion further aggravates the situation as IL-21 becomes limited, especially in the liver where HCV-specific CD8 T cells become dysfunctional and more susceptible to apoptosis. Finally, to counteract liver inflammation, Tregs are induced and act to further inhibit expansion of Th17 CD4 T cells while producing Gal-9 which can lead to the apoptosis of Tim-3 expressing CD4 and CD8 T cells. Polymorphism in the CD39 gene and the capacity of different HLA alleles to up-regulate inhibitory receptors may be the tipping point in determining the level of exhaustion and the outcome of infection in HCV patients (Figure 8).

## Materials and Methods

### Ethics statement, study subjects and clinical follow-up

HCV acutely infected subjects were recruited among high-risk HCV-seronegative IDUs participating in the Montreal Hepatitis C Cohort (HEPCO) at St. Luc hospital of the Centre Hospitalier de l'Université de Montréal as previously described [40], [78]. This study was approved by the institutional ethics committee *(SL05.014)*. All participants signed informed consent forms upon enrolment and experiments were performed in accordance with the Declaration of Helsinki. Participants were followed at scheduled 3-month intervals with a maximum duration of 22 weeks between visits. Acute infection was defined as either (i) detection of positive HCV RNA in the absence of HCV antibodies, followed by sero-conversion; (ii) a positive HCV antibody test following a previous negative test in the presence of positive HCV RNA; or (iii) a positive HCV antibody and RNA test within 3 months of a high-risk exposure. All patients tested negative for HIV and HBV. The estimated time of infection at recruitment was defined as the median time (in weeks) between the last negative test and first positive HCV RNA or antibody test. Spontaneous viral resolution (SR, n = 13) or chronic infection (CI, n = 24) was defined as the absence or the presence of HCV RNA at 24 weeks post-estimated time of infection (PEI), respectively. Two time points were studied for acutely infected patients: early acute (10 wks±1 wk PEI) and late acute (30 weeks±6 weeks PEI). Two additional categories of patients were studied: long-term HCV spontaneous resolvers (R) (n = 8), defined as HCV RNA negative and antibody positive at two consecutive tests >60 days apart, and long-term HCV chronically infected patients (C) (n = 27), defined as HCV RNA and antibody positive at recruitment with no prior negative test data. Healthy controls (n = 18) were also studied. The characteristics and demographics of the study participants are summarized in Table 1. HLA typing was performed as previously described [79].

### HCV RNA testing and quantification

Qualitative HCV RNA tests were performed using an automated COBAS Ampliprep/COBAS Amplicor HCV test, version 2.0 (sensitivity, 50 IU/ml) (Roche Molecular Systems, Inc, Branchburg, NJ). HCV genotyping was done using standard sequencing for the NS5B region. Both tests were performed by the Laboratoire de Santé Publique du Québec (St.-Anne-de-Bellevue, QC, Canada) as part of the clinical follow- up of patients.

### ELISA assays

The concentrations of IL-17A and IL-21 were determined in plasma samples collected in EDTA and culture supernatants using commercial ELISA kits (eBioscience, San Diego, CA), according to the manufacturer's protocols. The lower detection limits of the kits are 4 and 31 pg/ml, respectively. The concentration of Gal-9 in plasma was determined using a commercial ELISA kit (Uscn Life Science Inc., Wuhan, China), according to the manufacturer's protocol. The lower detection limit of the kit is 7.8 pg/ml.

### Peptides and peptide-HLA class I tetramers

Peptides were synthesized by Sheldon biotechnology Centre, McGill University, (Montreal, QC, Canada). MHC class I tetramers were synthesized by either the National Immune Monitoring Laboratory (NIML) (Montréal, QC, Canada) or the NIH Tetramer Core Facility (Emory University, Atlanta, GA) and are as follows: HLA-A1-restricted HCV NS3 peptide aa 1436 to 1444 (ATDALMTGY) (A1/NS3-1436), HLA-A2-restricted HCV NS3 peptide aa 1073 to 1081 (CINGVCWTV) (A2/NS3-1073), HLA-A2-restricted HCV NS3 peptide aa 1406 to 1415 (KLVALGINAV) (A2/NS3-1406), HLA-A2-restricted HCV NS5b peptide aa 2594 to 1415 (A2/NS5b-2594), HLA-B7-restricted HCV core peptide aa 41 to 49 (GPRLGVRAT) (B7/core-41), HLA-B8-restricted HCV NS3 peptide aa 1395 to 1403 (HSKKKCDEL) (B8/NS3-1395) and HLA-B27-restricted HCV NS5b peptide aa 2841 to 2849 (ARMILMTHF) (B27/NS5b-2841).

### Flow cytometry antibodies and reagents

Directly conjugated antibodies against the following surface molecules were used: CD4- PerCP (clone SK3), CD8- APC-H7 (clone SK1), CD25-PE or -PE-Cy7 (clone M-A251), CD26- FITC (Clone L272), CD161-PE-Cy5 (clone DX12), CCR5-FITC (clone 2D7), CCR6-PE (clone 11A9) and PD-1-FITC (clone MIH4) (all from BD Biosciences, San Jose, CA); CD39-PE or –PECy7 (clone eBioA1), CD127-eFluor 450 (clone eBioRDR5), CD160-Alexa 647 (clone BY55) (eBioscience); CD3- ECD (clone UCHT1) (Beckman Coulter, Marseille, France); Tim-3-PE or –PerCP (clone 344823) and CXCR6-PE (clone 56811) (R&D Systems, Minneapolis, MN). The following intracellular antibodies were used: CTLA-4-APC (clone BNI3), caspase-3-Alexa 647 (clone C92-065), TNF-α-Alexa 700 (clone Mab11) and IFN-γ-FITC (clone 25723) all from BD Biosciences; IFN-γ-eFluor 450 (clone 4SB3), IL-17A-Alexa 647 (clone eBio64DEC17), IL-21-PE (clone eBio3A3-N2) and FoxP3-Alexa 488 (clone PCH101) (eBioscience). Live cells were identified using Aqua Live/Dead Fixable Dead Cell Stain Kit according to the manufacturer's protocol (Life Technologies, Burlington, ON). “Fluorescence minus one” control stains were used to determine background levels of staining. Multiparameter flow cytometry was performed using a standard BD LSR II instrument equipped with blue (488 nm), red (633 nm), and violet (405 nm) lasers (BD Biosciences,) to systematically perform 11-9 color staining using the FACSDiva software (Version 5.0.3) (BD Biosciences). Compensation was performed with single fluorochromes and BD CompBeads (BD Biosciences). Biexponential transformation was applied during the analysis of data files using FlowJo software, version 9.4.11 for Mac (Tree Star, Inc., Ashland, OR).

### Cell separation

Tregs were isolated using CD25 Microbeads II (Miltenyi Biotech, Auburn, CA, USA) and the purity was assessed by flow cytometry. CD4 T cells were purified by negative selection using a CD4 T Cell Isolation kit II (Miltenyi Biotech), according to the manufacturer's instructions.

### Sorting experiments

All sorts were performed using the FACS Aria II Instrument (BD Biosciences) employing FACSDiva software (Version 5.0.3). For Th17 progenitor cells, purified CD4 T cells were labeled and sorted according to viability and the expression of CD3, CD4, CD127, CD161, CCR6 and CD26. CD127^low^ Tregs were excluded from the sort by gating on CD127^high^ CD4 T cells. Two populations were collected: CD127^high^CD161^neg^ or CD127^high^CD161^high^CCR6^hi^CD26^hi^ CD4 T cells. Sorted cells were stimulated for 48 hours with anti-CD3/anti-CD28 before mRNA extraction and RT-PCR. For the purification of Tregs, CD4 T cells were isolated, labeled and sorted according to viability and the expression of CD3, CD4, CD127, CD25 and CD39. Three populations were collected: effector T cells (CD25^neg^CD127^hi^), CD39^+^ Tregs (CD127^low^CD25^high^CD39^+^) and CD39^neg^ Tregs (CD127^low^CD25^high^CD39^neg^). Sorted cells were washed, and immediately lysed to extract mRNA as described below or fixed using a Foxp3 Staining Buffer Set (eBiosciences) to perform the intracellular staining of FoxP3 and CTLA-4, according to the manufacturer's instructions.

### Multiparametric phenotypic characterization of HCV-specific T cells

All flow cytometry assays were performed on cryo-preserved samples. For the phenotype analysis, 2×10^6^ PBMCs were stained with freshly prepared tetramer-PE for 30 minutes at room temperature and washed in fluorescence-activated cell sorting (FACS) buffer (1× phosphate-buffered saline [PBS], 1% fetal bovine serum [FBS], 0.02% NaN_3_). Samples were then stained with surface antibodies for 30 minutes at 4°C, washed twice in FACS buffer, and fixed in FACS Fix buffer (1× PBS, 1% formaldehyde).

### Intracellular cytokine staining

Cells were stimulated with PMA/ionomycin (50 ng/ml and 1 µg/ml, respectively). Following 2 hours of stimulation, 5 µg/ml of brefeldin A and 5 µg/ml of monensin sodium salt were added, and cells were incubated for a total of 16 hours. Cells were washed with FACS buffer, stained for viability and cell surface antigens, fixed and permeabilized using a FoxP3 buffer solution (eBioscience). Then, the cells were stained with anti-IL-17A, anti-IL-21 and anti-IFN-γ antibodies for 30 minutes and washed twice in Perm buffer (eBioscience). For the analysis, cells were gated on viable CD3^+^ CD8^−^ T cells.

### CFSE proliferation assays

CD25-depleted PBMCs were re-suspended in PBS at 20×10^6^ cells/ml and stained with 0.5 µM CFSE (Life Technologies, Burlington, ON, Canada) for 8 minutes at room temperature. The reaction was stopped with human serum. Cells were washed three times in PBS and then re-suspended at 2×10^6^ cells/ml in warm RPMI (Life Technologies), 10% FCS (R-10) medium. CFSE-labeled cells were stimulated for 6 days with or without 1 µg/ml of the HCV-recombinant proteins NS3 and NS4 (Feldan, Quebec, QC, Canada) in the presence of 200 ng/ml of anti-CD28/-CD49d (Fastimmune, BD bioscience) at 37°C and 5% CO_2_. Recombinant human IL-2 (20 IU/ml) (NIH AIDS Research and Reference Reagent Program, Germantown, MD) was added on day 3. Some assays were performed in the presence of blocking antibodies against PD-L1 (clone MIH1, eBioscience), CTLA-4 (Clone BNI3, BD biosciences) and Tim-3 (Clone F38-2E2, Biolegend) at 10 µg/ml each or in the presence of IgG1 and IgG2a isotype control antibodies. On day 6, cells were directly stained with surface antigens as described above or stimulated by PMA/ionomycin to assess cytokine secretion by HCV-specific T cells, identified by CFSE^low^ expression.

### HCV epitope-specific proliferation assay

PBMCs were stimulated with 10 µg/ml of the cognate HCV peptide for 6 days in the presence or absence of IL-21 (20 ng/ml). HCV-specific cells were identified as HCV tetramer^+^CD8^+^ T cells. Fold expansion was calculated by dividing the frequency of HCV tetramer^+^CD8^+^ T cells after *in vitro* expansion by the frequency measured directly *ex vivo.*

### *Ex vivo* apoptosis assay

PBMCs were incubated for 4 hours with varying concentrations of Gal-9 or Gal-3 (1 µM each) in R-10. Cells were then stained for surface markers and the specific HCV-tetramers followed by Annexin V staining using Annexin V Apoptosis Detection Kit I (BD biosciences). Cells were fixed and permeabilized with FoxP3 specific buffer (eBioscience) followed by intracellular staining with the anti-caspase-3 antibody.

### Relative quantification of Th17 specific transcription factor mRNA by real-time quantitative reverse transcription polymerase chain reaction (RT-PCR)

Total RNA was extracted using Real-Time Ready Cell Lysis kit (Roche, Laval, QC, Canada) according to the manufacturer's instructions. Reverse transcription was performed using Transcriptor Universal cDNA Master (Roche). Real-time PCR amplification was performed using commercial primers (Qiagen, Toronto, ON, Canada) for RORc (Assay ID QT00097888, Gene bank accession number NM_005060) or c-MAF (QT00023618, NM_005360) (Applied Biosystems, Foster City, CA, USA) in combination with the LightCycler 480 SYBR Green I Master (Roche). Transcription factor gene expression was quantified with the Advanced Relative Quantification method provided by the manufacturer and normalized with 28S mRNA expression. Quantitative PCR was performed using a LightCycler 480 detection system (Roche).

### Relative quantification of Galectin-9 mRNA by RT-PCR

Total RNA was extracted and reverse transcribed as described above. Real-time PCR amplification was performed using the pre-developed assay-on-demand gene expression set for the Gal-9 gene (*LGALS9*) (Assay ID Hs00247135_m1, Gene bank accession number NM_009587.2, Applied Biosystems, Foster City, CA, USA) and the Human 18S endogenous control (HS_99999901, Gene bank accession number X03205.1, Applied Biosystems) using the Taqman Universal PCR Master Mix. The quantification of Gal-9 mRNA expression was calculated with the absolute method provided by the manufacturer and expressed in Tregs relative to the expression in CD127^hi^CD25^neg^ effector T cells. Quantitative PCR was performed using a LightCycler 480 detection system (Roche).

### Small interfering RNA transduction

For the silencing of *LGALS9*, we performed RNA interference experiments on isolated Treg cells according to the manufacturer's protocol by using *Silencer* siRNA Transfection II Kit (Ambion, Applied Biosystems). Knockdown of *LGALS9* in Treg cells was confirmed by qRT-PCR as shown in Supplementary Figure S5. Control Treg cells received a scrambled siRNA. Cells were allowed to recover in R-10 complete media (RPMI, 10% FCS) at 37°C for 5 hours then used in the co-culture assay.

### Co-culture assay

The combined CFSE/ICS assays were performed as described above in the presence of Tregs added at a ratio of 1∶4 (Tregs∶CD25-depleted CFSE labeled PBMCs). The percentage inhibition of cytokine production upon addition of Tregs to the stimulated PBMC cultures was calculated using the following formula: (percentage CFSE^low^ cytokine^+^CD8^neg^ T cells in absence of Tregs – percentage CFSE^low^ cytokine^+^CD8^neg^ T cells in presence of Tregs)/percentage CFSE^low^ cytokine^+^CD8^neg^ T cells in absence of Tregs×100.

### Statistical analysis

All analyses were performed using GraphPad Prism version 5.0 (GraphPad Software, San Diego, CA, USA). The Mann-Whitney U rank sum test was performed to compare the median values between two groups. The Wilcoxon signed rank test was performed to compare the median values between two paired groups. Correlations were determined by the Spearman rank test. P-values<0.05 were considered significant.

## Supporting Information

Figure S1 **Enhanced proliferation of HCV-specific CD4 T cells during acute resolving HCV infection.** (**A**) Schematic representation of CFSE/ICS assay. PBMCs were depleted of CD25^+^ cells then labelled with CFSE and stimulated with HCV NS3 or NS4 recombinant proteins (1 µg/ml) during 6 days. Un-stimulated and SEB-stimulated cells were used as negative and positive controls, respectively. At the end of day 6, cells were stimulated with PMA/ionomycin for 18 hours with addition of brefeldin A and monensin to reveal their cytokine expression pattern. HCV-specific CD4 T cells were identified by the dilution of CFSE coupled with the up-regulation of CD25 within the CD3^+^CD8^neg^ lymphocyte gate. (**B**) Representative figure of CFSE proliferation assay in an SR *vs* a CI patient. CD25-depleted PBMCs were stained with CFSE and stimulated with HCV NS3 or NS4 recombinant proteins (1 µg/ml) during 6 days. Un-stimulated and SEB-stimulated cells were used as negative and positive controls, respectively. Cells were gated on CD3^+^CD8^neg^ lymphocytes. Antigen-specific T cells were identified as CD25^high^CFSE^low^ CD4 T cells. (**C**) Stimulation Index (SI) of HCV-specific CD4 T cells at the indicated time points was calculated using the following formula: % CD25^high^CFSE^low^ (HCV specific)/% CD25^high^CFSE^low^ (Un-stimulated).
(TIF)

Figure S2 **Representative figure of combined CFSE proliferation/intracellular cytokine staining (ICS) assay.** To characterize the cytokine profile of HCV-specific CD4 T cells, CD25-depleted PBMC from patients with acute HCV were stained with CFSE and stimulated with 1 µg/ml of HCV-recombinant proteins (NS4). After 6 days of culture, cells were washed and re-stimulated with PMA/ionomycin in presence of brefeldin A/monensin to reveal the cytokine profile by ICS as described in Materials and Methods and Figure S1A. Cells were gated on viable CD3^+^CD8**^neg^** lymphocytes for analysis of the percent of cytokine^+^CFSE^low^ cells.
(TIF)

Figure S3 **Increased frequency of Th17 cells during acute resolving HCV.** (**A**) Increased frequency of IL-21-secreting Th17 cells in SR patients during acute infection. PBMCs from HCV infected patients collected at pre-infection and late acute HCV as well as PBMCs from long-term chronic patients were stained to evaluate the frequency of Th17 T cells defined as CD161^high^CCR6^+^CD26^+^ CD4 T cells. For pre-infection samples, grey symbols represent SR and black symbols represent CI patients. (**B**) Representative FACS plot for the identification of IL-21-secreting Th17 cells as CD161^high^CCR6^+^CD26^+^ CD4 T cells. Purified CD4 T cells were first gated on CD127^high^ cells to exclude Tregs and then gated on CD161^high^ cells and then based on co-expression of CD26 and CCR6 to define the Th17 population. (**C**) Characterization of IL-21-producing Th17 cells by specific expression of Th17 transcription factors. CD161^neg^ and CD161^high^CCR6^+^CD26^+^ CD4 T cells were sorted from HCV long-term resolvers (R) (n = 5) or chronic (C) (n = 4) patients. Cells were stimulated for 48 hours with anti-CD3/anti-CD28 and gene expression of RORc or c-MAF was evaluated using specific commercial primers and normalized to 28S mRNA expression.
(TIF)

Figure S4 **Reduced proliferative capacity of Tim-3^high^ cells.** PBMCs from acute HCV patients were stimulated with their cognate peptide epitopes corresponding to the HCV MHC class I tetramers used as described in Materials and Methods. Patients were classified according to Tim-3 expression on HCV tetramer^+^ CD8^+^ T cells as: Tim-3^neg^ (open circles), Tim-3^low^ (grey circles) and Tim-3^high^ (closed circles). Data is presented as the frequency of HCV tetramer^+^ CD8^+^ T cells directly *ex-vivo* and after *in vitro* stimulation and expansion by the cognate peptide.
(TIF)

Figure S5 **Co-culture assay.** (**A**) Representative model of Treg co-culture in CFSE/ICS assay. Combined CFSE/ICS assays were performed as described in Materials and Methods and Figure S1 in the presence of Tregs added at a ratio of 1∶4 (Tregs∶CD25-depleted CFSE-labeled PBMC). Tregs were transduced with scrambled, GAPDH or *LGALS9* siRNA. (**B**) Silencing of Galectin-9 expression in regulatory T cells from HCV chronic patients. The efficiency of knockdown of Gal-9 expression in Tregs following transfection of *LGALS9* siRNA was assessed by quantitative RT-PCR. Purified CD25^+^ CD4 T cells were transfected with scrambled, GAPDH or *LGALS9* siRNA. The mRNA was isolated and GAPDH or *LGALS9* gene expression was normalized to 18S mRNA expression (p<0.01).
(TIF)
