# Supplementary figures and images for: Galectin-9 and IL-21 Mediate Cross-regulation between Th17 and Treg Cells during Acute Hepatitis C
Source: PLoS Pathog. 2013 Jun 20;9(6):e1003422. doi: 10.1371/journal.ppat.1003422 (PMC3688567; doi:10.1371/journal.ppat.1003422)

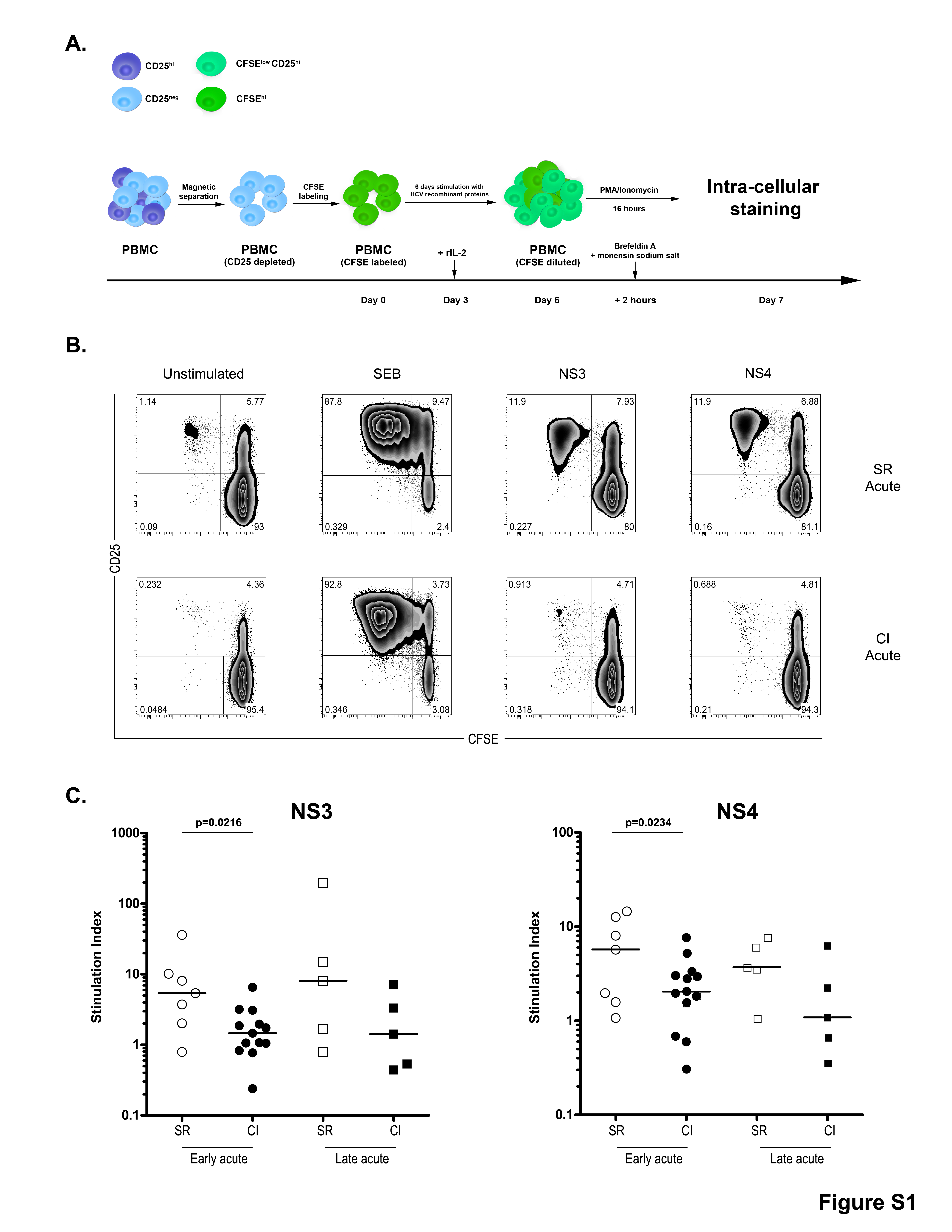

Supplement: Figure S1 — Enhanced proliferation of HCV-specific CD4 T cells during acute resolving HCV infection. (A) Schematic representation of CFSE/ICS assay. PBMCs were depleted of CD25+ cells then labelled with CFSE and stimulated with HCV NS3 or NS4 recombinant proteins (1 µg/ml) during 6 days. Un-stimulated and SEB-stimulated cells were used as negative and positive controls, respectively. At the end of day 6, cells were stimulated with PMA/ionomycin for 18 hours with addition of brefeldin A and monensin to reveal their cytokine expression pattern. HCV-specific CD4 T cells were identified by the dilution of CFSE coupled with the up-regulation of CD25 within the CD3+CD8neg lymphocyte gate. (B) Representative figure of CFSE proliferation assay in an SR vs a CI patient. CD25-depleted PBMCs were stained with CFSE and stimulated with HCV NS3 or NS4 recombinant proteins (1 µg/ml) during 6 days. Un-stimulated and SEB-stimulated cells were used as negative and positive controls, respectively. Cells were gated on CD3+CD8neg lymphocytes. Antigen-specific T cells were identified as CD25highCFSElow CD4 T cells. (C) Stimulation Index (SI) of HCV-specific CD4 T cells at the indicated time points was calculated using the following formula: % CD25highCFSElow (HCV specific)/% CD25highCFSElow (Un-stimulated). (TIF) [file ppat.1003422.s001.tif]

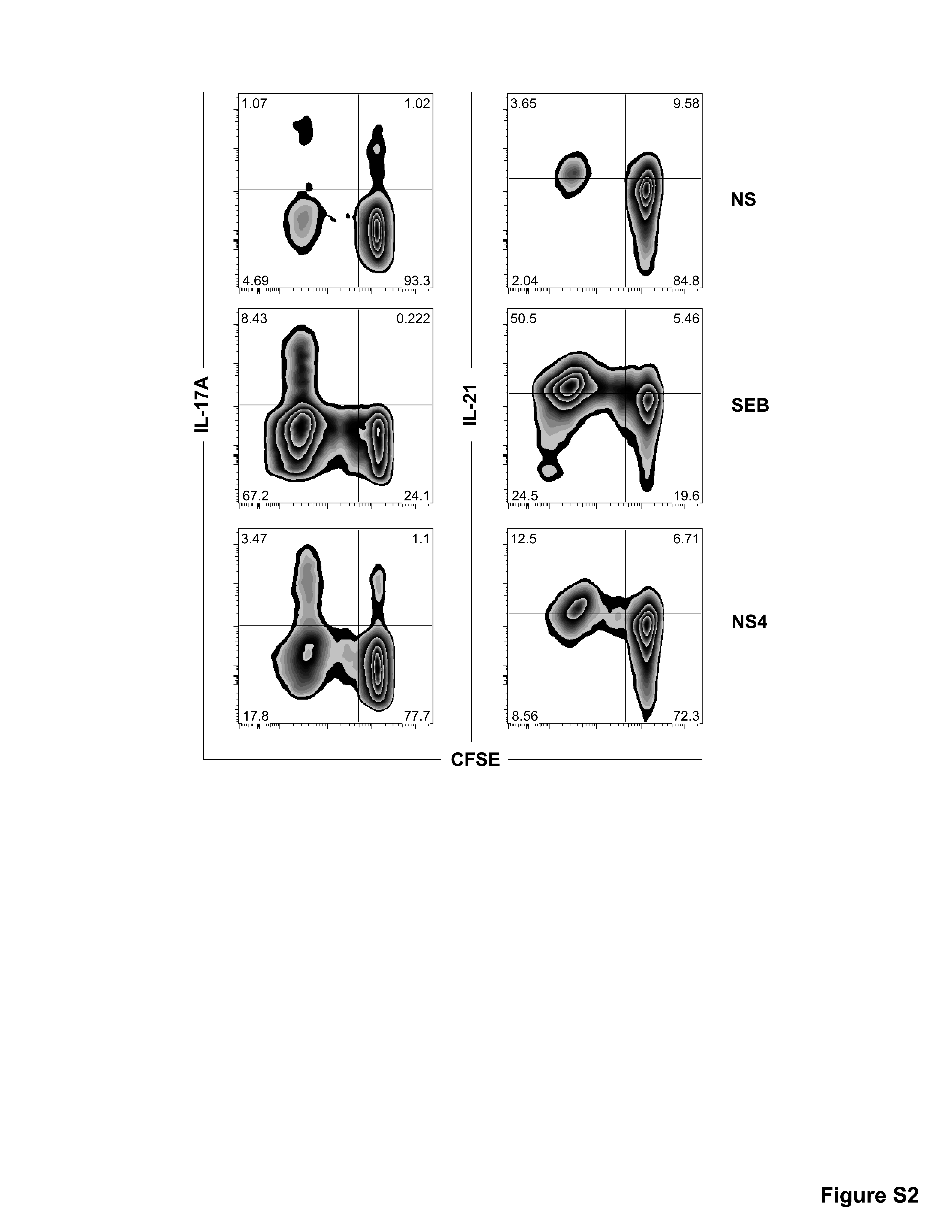

Supplement: Figure S2 — Representative figure of combined CFSE proliferation/intracellular cytokine staining (ICS) assay. To characterize the cytokine profile of HCV-specific CD4 T cells, CD25-depleted PBMC from patients with acute HCV were stained with CFSE and stimulated with 1 µg/ml of HCV-recombinant proteins (NS4). After 6 days of culture, cells were washed and re-stimulated with PMA/ionomycin in presence of brefeldin A/monensin to reveal the cytokine profile by ICS as described in Materials and Methods and Figure S1A. Cells were gated on viable CD3+CD8neg lymphocytes for analysis of the percent of cytokine+CFSElow cells. (TIF) [file ppat.1003422.s002.tif]

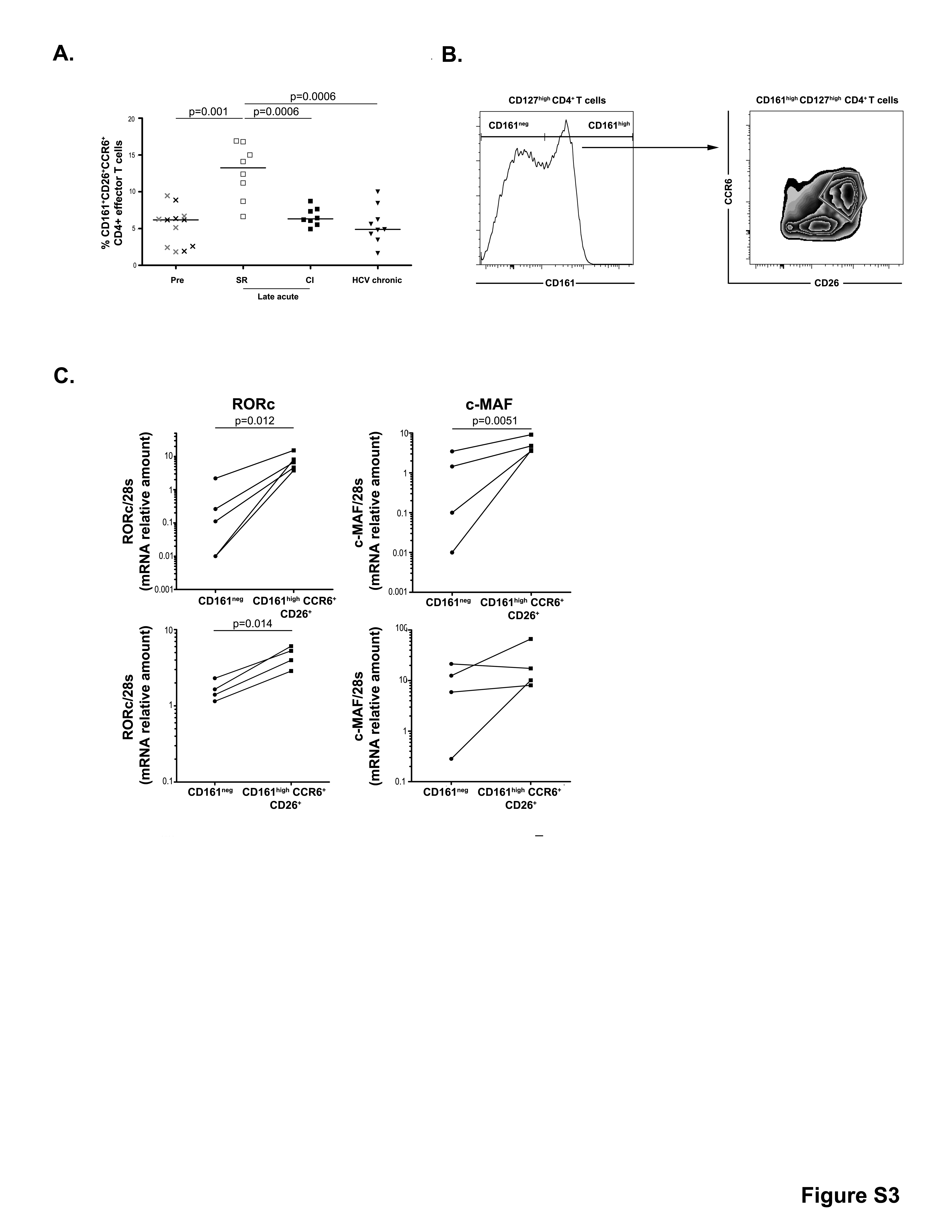

Supplement: Figure S3 — Increased frequency of Th17 cells during acute resolving HCV. (A) Increased frequency of IL-21-secreting Th17 cells in SR patients during acute infection. PBMCs from HCV infected patients collected at pre-infection and late acute HCV as well as PBMCs from long-term chronic patients were stained to evaluate the frequency of Th17 T cells defined as CD161highCCR6+CD26+ CD4 T cells. For pre-infection samples, grey symbols represent SR and black symbols represent CI patients. (B) Representative FACS plot for the identification of IL-21-secreting Th17 cells as CD161highCCR6+CD26+ CD4 T cells. Purified CD4 T cells were first gated on CD127high cells to exclude Tregs and then gated on CD161high cells and then based on co-expression of CD26 and CCR6 to define the Th17 population. (C) Characterization of IL-21-producing Th17 cells by specific expression of Th17 transcription factors. CD161neg and CD161highCCR6+CD26+ CD4 T cells were sorted from HCV long-term resolvers (R) (n = 5) or chronic (C) (n = 4) patients. Cells were stimulated for 48 hours with anti-CD3/anti-CD28 and gene expression of RORc or c-MAF was evaluated using specific commercial primers and normalized to 28S mRNA expression. (TIF) [file ppat.1003422.s003.tif]

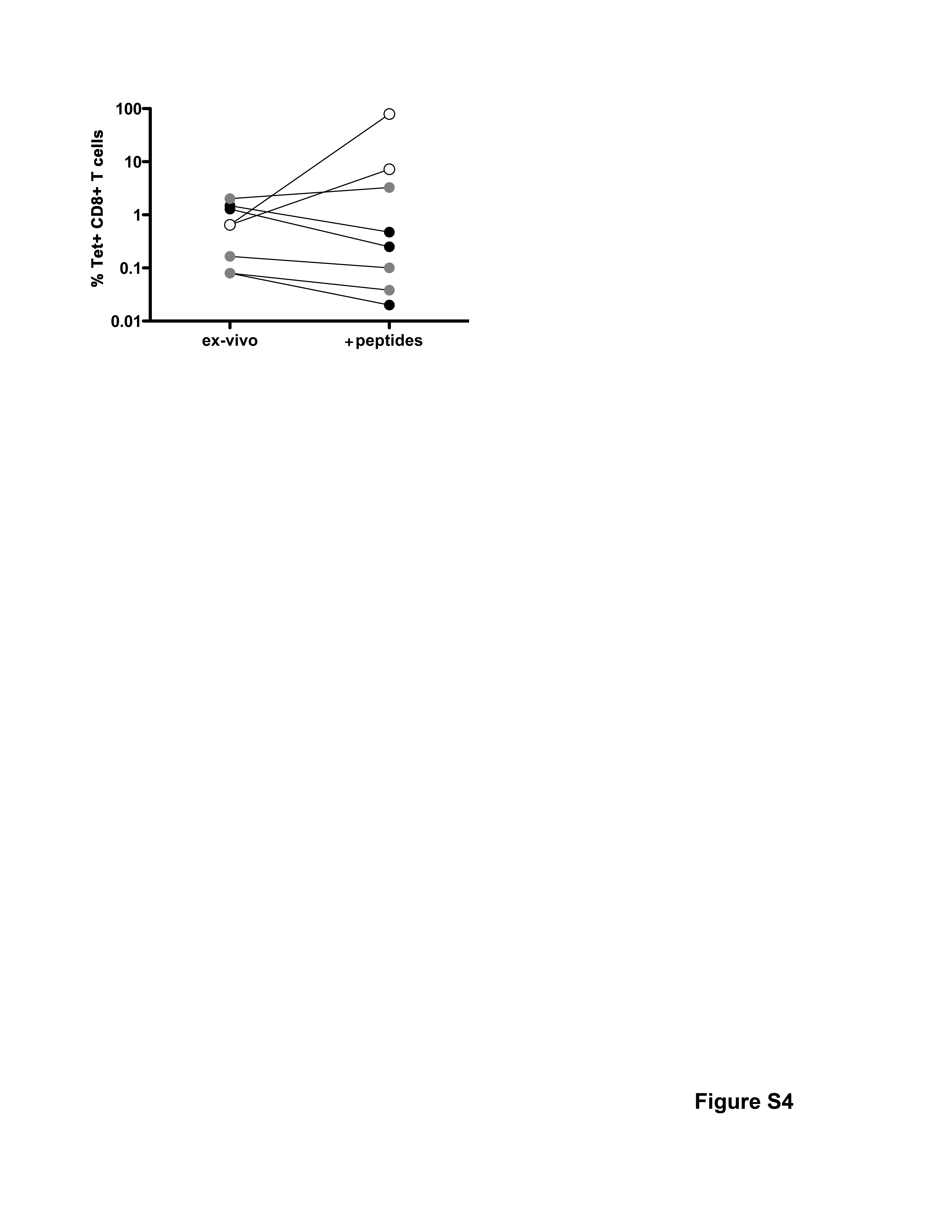

Supplement: Figure S4 — Reduced proliferative capacity of Tim-3high cells. PBMCs from acute HCV patients were stimulated with their cognate peptide epitopes corresponding to the HCV MHC class I tetramers used as described in Materials and Methods. Patients were classified according to Tim-3 expression on HCV tetramer+ CD8+ T cells as: Tim-3neg (open circles), Tim-3low (grey circles) and Tim-3high (closed circles). Data is presented as the frequency of HCV tetramer+ CD8+ T cells directly ex-vivo and after in vitro stimulation and expansion by the cognate peptide. (TIF) [file ppat.1003422.s004.tif]

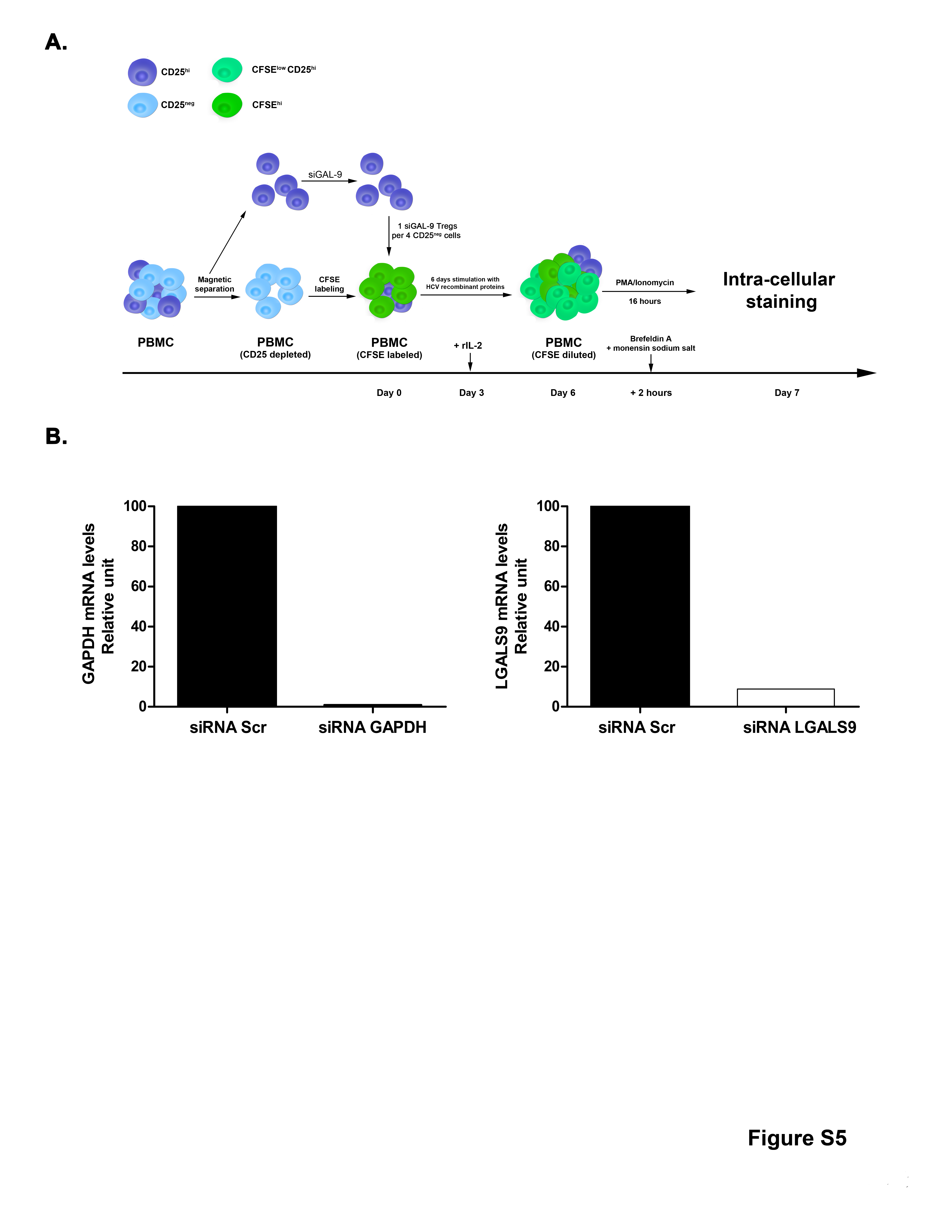

Supplement: Figure S5 — Co-culture assay. (A) Representative model of Treg co-culture in CFSE/ICS assay. Combined CFSE/ICS assays were performed as described in Materials and Methods and Figure S1 in the presence of Tregs added at a ratio of 1∶4 (Tregs∶CD25-depleted CFSE-labeled PBMC). Tregs were transduced with scrambled, GAPDH or LGALS9 siRNA. (B) Silencing of Galectin-9 expression in regulatory T cells from HCV chronic patients. The efficiency of knockdown of Gal-9 expression in Tregs following transfection of LGALS9 siRNA was assessed by quantitative RT-PCR. Purified CD25+ CD4 T cells were transfected with scrambled, GAPDH or LGALS9 siRNA. The mRNA was isolated and GAPDH or LGALS9 gene expression was normalized to 18S mRNA expression (p<0.01). (TIF) [file ppat.1003422.s005.tif]
